# Head hemodynamics and systemic responses during auditory stimulation

**DOI:** 10.14814/phy2.15372

**Published:** 2022-07-03

**Authors:** Vanesa Muñoz, José A. Diaz‐Sanchez, Manuel Muñoz‐Caracuel, Carlos M. Gómez

**Affiliations:** ^1^ Human Psychobiology Laboratory, Experimental Psychology Department University of Sevilla Sevilla Spain

**Keywords:** auditory stimulation, electrodermal activity, fNIRS, heart rate, pulse signal, pulse transit time

## Abstract

The present study aims to analyze the systemic response to auditory stimulation by means of hemodynamic (cephalic and peripheral) and autonomic responses in a broad range of auditory intensities (70.9, 77.9, 84.5, 89.5, 94.5 dBA). This approach could help to understand the possible influence of the autonomic nervous system on the cephalic blood flow. Twenty‐five subjects were exposed to auditory stimulation while electrodermal activity (EDA), photoplethysmography (PPG), electrocardiogram, and functional near‐infrared spectroscopy signals were recorded. Seven trials with 20 individual tones, each for the five intensities, were presented. The results showed a differentiated response to the higher intensity (94.5 dBA) with a decrease in some peripheral signals such as the heart rate (HR), the pulse signal, the pulse transit time (PTT), an increase of the LFnu power in PPG, and at the head level a decrease in oxygenated and total hemoglobin concentration. After the regression of the visual channel activity from the auditory channels, a decrease in deoxyhemoglobin in the auditory cortex was obtained, indicating a likely active response at the highest intensity. Nevertheless, other measures, such as EDA (Phasic and Tonic), and heart rate variability (Frequency and time domain) showed no significant differences between intensities. Altogether, these results suggest a systemic and complex response to high‐intensity auditory stimuli. The results obtained in the decrease of the PTT and the increase in LFnu power of PPG suggest a possible vasoconstriction reflex by a sympathetic control of vascular tone, which could be related to the decrease in blood oxygenation at the head level.

## Graphical abstract

The results found in the present study could indicate the presence of an autonomic systemic response to high sound intensity. The results obtained in the decrease of the pulse transit time suggest a possible vasoconstriction reflex.

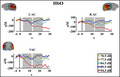

## 1 INTRODUCTION

The autonomic nervous system (ANS) plays an important role in the control of physiological systems. Its main function is to maintain homeostasis and regulate body functions, through the autonomic reflexes and control centers. The ANS has afferent and efferent parts that allow transmitting sensory information by employing vegetative fibers that receive the sensory inputs, process the signals, and send a specific response (Cardinali, 2018; Ernst, 2014; Wehrwein et al., 2016). This system has been divided into three branches, sympathetic (SNS), parasympathetic (PNS), and enteral. The regulation and adaptation processes carried out by the ANS are important in the face of environmental changes and threats, and allow the organism to respond and adapt to stressful or intense stimuli such as high‐intensity sounds.

In this sense, the amygdala plays an important role in the interpretation of the environmental threats such as images or sounds, generating stress responses through the stimulation of the SNS, with projections to the hypothalamus and brainstem, which are responsible for the regulation of the metabolic processes and functioning of the ANS. Stress responses generally release an increase in epinephrine, which elicits a physiological response in effector organs observed as an increase in heart rate (HR) and blood pressure (BP), the latter due to blood vessels vasoconstriction (Jarczewski et al., 2019). The process of the ANS regulation, in the face of threats or stressors, could be different for central and peripheral blood vessels, therefore could be useful to measure autonomic variables that indicate peripheral or systemic changes such as HR, heart rate variability (HRV), photoplethysmography (PPG), electrodermal activity (EDA), and pulse transit time (PTT), together with measures of central variables as functional near‐infrared spectroscopy (fNIRS).

One of the ANS signals, which has usually been studied in the face of a stressful environmental or experimental condition, is the EDA. The sweating process is the physiological response linked to this measurement, is usually induced by stressful events, and is controlled by sympathetic cholinergic fibers. In the presence of SNS activity, the eccrine sweat glands increase the production of sweat, and this change affects the capacity of the skin to conduct electricity (Chang et al., 2012). This skin conductance is composed of two components: Tonic and phasic, which represent different relations to the stimulus, the former being related to the skin conductance level (SCL) and the latter to the skin conductance response (SCR). The phasic component (SCR) is represented by a fast‐changing component and is defined by a skin conductance transient arising within a predefined window (Greco et al., 2016). In turn, the tonic electrodermal component (SCL) represents the baseline level of the signal, changes in the SCL are thought to reflect general changes in autonomic arousal (Braithwaite et al., 2013). Previous studies have shown a close relationship between sympathetic system activation and increased sweating which makes EDA a suitable method for accessing SNS responses during psychological stress, such as during high‐intensity auditory stimulation (Kato et al., 2014). This has led to this measure as one of the most studied in auditory paradigms, not just in healthy subjects (Kato et al., 2014) but also in a wide variety of disorders such as anxiety, autism, or menopausal women where an increase in skin conductance (SCR and/or SCL) has been found with an increase in sound intensity (Bakker et al., 2009; Bharath et al., 2020; Chang et al., 2012; Kishan et al., 2018; Pfeiffer et al., 2019).

Another relevant physiological response for the assessment of the ANS activity is the analysis of HR and HRV, taking into account that the HR is under continuous control of the SNS and PNS, in both stress and basal conditions. The duration of the cardiac cycle and its fluctuations are largely mediated by the ANS, which regulates the sinus node discharge rhythm (Hayano, 2017). The analysis of HR to auditory stimulation has been widely studied and has led to extensive discussions on the diversity of responses to different auditory intensities. Studies since Sokolov (1963), Sokolov et al. (2002), Graham and Clifton (1966), and more recently Vila et al. (2007), have found patterns of acceleration or deceleration, and in other cases of both when high‐intensity auditory stimuli are presented. The debate with these seemingly disparate results arises with the approach of defense and orienting reflexes, which have been widely discussed. The use of various classes of stimuli and different rise times in the auditory stimuli possibly have an impact on the type of response elicited, whether it is a cardiac acceleration, deceleration, or both (Barry & Maltzman, 1985). An important study in this regard, by Shoushtarian et al. (2019) in which the fNIRS signal was used for the evaluation of cardiac measurements, found a decrease in HR for sound levels between 15 and 40 dB and an increase for 65 and 90 dB. Furthermore, subpopulations of subjects have shown decelerative atypical HR responses during startle reflex (Chou et al., 2014).

Another measure of cardiac response is HRV, which is analyzed in the time domain by the changes in the time intervals between adjacent R peaks in the electrocardiogram (ECG) signal and by changes in the power spectral density of the ECG signal in the frequency domain. It has an important regulatory role in adaptation to environmental changes, by stimulating and regulating some vascular components, being a reflection of the regulation of autonomic balance (Minarini, 2020). There are three types of measurements to analyze the HRV: time‐domain, frequency‐domain, and nonlinear measurements. In the time domain index, the most common measure is the standard deviation of RR intervals, the other commonly used alternative measures are based on the differences between RR intervals, such as the root mean square of successive RR interval differences (RMSSD) and the number of pairs of adjacent RR intervals that differ by more than 50 ms (NN50 count). Considering that the calculation uses the RR interval differences, these measures reflect mainly the high‐frequency (HF) variations in HRV and the PNS activity (Kiyono et al., 2017; Kuusela, 2012).

In turn, the frequency‐domain index can be useful to obtain more detailed information about the dynamics and frequency components of the HRV, employing analysis methods such as power spectral density (PSD), which decomposes the signal into its frequency components. This measurement has been widely employed to assess sympathetic activity, taking into account that it seems that low frequencies are associated with SNS activity. According to this approach, an increase in the LF indicates an increase in sympathetic activity, and a higher value in LF/HF ratio could indicate a shift in the sympathetic‐vagal balance toward sympathetic predominance (Kiyono et al., 2017). Recently, however, the issue of accessing sympathetic activity through the study of LF has been quite controversial; some studies have found results that contradict the assumption that this band reflects purely sympathetic activity and could also be affected by the PSN. (Ernst, 2014; Reyes del Paso et al., 2013). Studies on HRV and auditory stimulation, both in time and frequency domain analysis have shown contradictory results, some of them have found an influence of sound intensity with changes at the level of sympathetic and parasympathetic balance, in specific populations or dependent on music style (do Amaral et al., 2015; Lee et al., 2010; Veternik et al., 2018). Nevertheless, the results remain inconclusive and the debate on the extent of autonomic measures of cardiac variability is still under discussion.

An additional measure of peripheral responses can be obtained using PPG. This measurement allows to record the changes in blood flow using infrared light that penetrates the tissue, usually in the finger, approximating blood volume by determining the amount of reflected light (Abay & Kyriacou, 2022). The PPG signal is used to measure blood volume pulse (BVP), which indicates variations in the volume of blood in vessels (Kushki et al., 2011). PPG reflects the changes in the blood volume and blood flow supplied to the region since represents the pumping action of the heart (Abay & Kyriacou, 2022). The PSD analysis of the PPG and its variability has been used as a way to study the regulation of peripheral vascular tone, taking into account that the BP oscillations are transmitted and projected into the PPG signal (Ishbulatov et al., 2020; Kiselev & Karavaev, 2020). Several studies have found that low‐frequency oscillations of PPG may be related to autonomic vascular control, and in turn reflect changes in sympathetic tone, whereas high frequencies have been associated with respiratory processes (Bernardi et al., 1996; Middleton et al., 2011). Recently some authors have emphasized its sensitivity in the study of some autonomic dysfunctions (Karavaev et al., 2021; Kiselev & Karavaev, 2020).

The PPG signal oscillates within the cardiac cycle, due to the systolic increase in tissue blood volume, resulting in reduced light transmission, thus this measurement can be used too as a complement to the ECG, showing changes in the cardiac cycle during environmental changes (Alian & Shelley, 2014). ECG and PPG signals are often used to calculate the PTT value, that is, the time it takes for the BP wave, caused by the systolic phase, to travel from the proximal artery to the distal location in the arterial trajectory (Feng et al., 2018; Mol et al., 2020). PTT is inversely proportional to the BP, when BP increases it causes vascular resistance in the arterial walls, increasing pulse wave velocity, and leading to a shorter PTT. Likewise, upon vasoconstriction, arterial stiffness increases and the PTT decreases. This measure is used to assess sympathetic activity in the face of an environmental change or stressful condition (Budidha & Kyriacou, 2014; Pollonini et al., 2015). Some studies have shown, in paradigms that activate the SNS as the ice water immersion, that the change in temperature leads to an increase in blood vessels resistance causing a decrease in PTT and pulse wave amplitude values while increasing BP and HR (Armañac‐Julián et al., 2019; Budidha & Kyriacou, 2014; Budidha & Kyriacou, 2019; van Velzen et al., 2016). In auditory paradigms, some studies have found a shorter PTT with auditory stimulation (Feger & Braune, 2005; Franco et al., 2002; Galland et al., 2007). This decrease in PTT is proposed as a vasoconstriction response produced by the sound stimulation perceived as a threat. However, to our knowledge, no study has included sound intensity.

All of the measurement tools presented above are generally used to access the peripheral and sympathetic responses in acute stress situations, however, at the cerebral level, the vascular stress response is less clear. The effect of the autonomic changes in the brain, currently, is a matter of discussion. The brain is one of the most perfused organs and has high metabolic requirements for its functioning. Blood flow in the brain needs to be constant and needs to preserve the intracranial pressure, in contrast to highly variable peripheral vascular responses, the arteries play a main role in the brain to maintain constant pressure and avoid abrupt changes that could affect brain function or even cause death (Ainslie & Brassard, 2014). The role of the SNS in the brain is controversial, some studies propose that its influence is minimal and modest (Laan et al., 2013). An approach to studying that influence noninvasively is through brain scanning techniques that measure cerebral hemodynamic activity, such as fNIRS (Sørensen et al., 2012).

fNIRS is an optical neuroimaging technique for measuring brain activity, based on optics and physiological concepts. This method uses a source of infrared light and a detector, usually with a standard separation of 3–4 cm. The infrared light with an appropriate wavelength (oxygenated hemoglobin <800 nm; deoxygenated hemoglobin >800 nm) can be absorbed by chromophores in the blood or scattered in the tissues, the attenuation of the incoming light is mainly due to the primary chromophore in the brain: hemoglobin, which is responsible for oxygen transport. This makes this method sensitive to changes in concentrations of the hemoglobin in the brain for oxygenated (HbO), deoxygenated (HbR), and total hemoglobin (HbT), and its advantages are relatively low cost, portability, and for auditory paradigms, its silent operation (Ferrari & Quaresima, 2012; Pinti et al., 2020). Its physiological functioning is based on the hemodynamic changes produced after brain activation. Considering the neurovascular coupling process, which explains that brain activity leads to an increase in oxygen consumption and therefore an increase in cerebral blood flow to compensate for the reduced availability of oxygen, in the so‐called hemodynamic response (Attwell et al., 2010).

Several fNIRS studies with auditory stimulation have found activation of the auditory cortex (Abla & Okanoya, 2008; Chen et al., 2015; Kochel et al., 2013; Minagawa‐Kawai et al., 2002; Rinne et al., 1999). However, most of the research relating to sound intensity has been developed with the fMRI technique (Brechmann et al., 2002; Hall et al., 2001; Hart et al., 2003; Langers et al., 2007; Röhl & Uppenkamp, 2012; Sigalovsky & Melcher, 2006), in which an increase in the BOLD response in the auditory cortex has been found related to the change in frequency and intensity of stimulation in both primary and secondary auditory cortex. On the other hand, with fNIRS the results have been contradictory and unclear, some studies have shown a positive correlation between fNIRS activation and sound intensity (Bauernfeind et al., 2018; Weder et al., 2018; Weder et al., 2020), but in other studies, this difference in activation is unclear (Chen et al., 2015; Muñoz‐Caracuel et al., 2021).

At this point, is important to emphasize that several studies have pointed out the influence of both cerebral and peripheral physiological interferences in the fNIRS signal (Caldwell et al., 2016; Kirilina et al., 2012; Tachtsidis & Scholkmann, 2016). Hence, it is important to take into account that, when analyzing the fNIRS signal, despite filtering, some physiological signals are superimposed on the neurovascular coupling frequencies, such as respiratory signals, vasoconstriction processes, BP, and therefore the sympathetic nervous system, and in many cases lead to false positives or negatives (Hocke et al., 2018). Recently, it has been proposed a method to reduce the influence of extracortical activity with the use of short channels, that is, channels with a detector source distance of 10–15 mm. Thus, these channels record the extracortical activity which is then subtracted from the overall signal recorded with the standard nirs channels (3–4 cm) (Brigadoi & Cooper, 2015) and therefore leading just the concentrations changes in the analyzed cortices. However, it has also been found in front of high intensities a decrease in HbO, HbT in central and peripheral areas (Muñoz‐Caracuel et al., 2021), which could imply that some extracortical responses would influence the cerebral hemodynamic response in a more generalized way and that the use of short channels may not minimize. In this sense, in a paradigm that elicits stress responses such as the presentation of a high‐intensity sound, the signal studied with fNIRS could indicate a systemic response of the ANS, at the central and peripheral levels.

Studies including peripheral signals such as EDA, HR, HRV, PPG, and PTT, as well as the recording of cerebral hemodynamic signals such as fNIRS, in auditory stimulation paradigms are uncommon in the literature. Nevertheless, studies that have addressed the issue on separate lines suggest sympathetic activity at high sound intensities, likewise, but less clearly, a differential reaction of the auditory cortex to the presentation of different sound intensities. Bearing this in mind, the present study takes as background a previous study performed in our laboratory, whose results are reported in the paper Muñoz‐Caracuel et al. (2021), in which a decrease in oxygenated hemoglobin was found for both short and standard channels of the auditory and prefrontal cortex, in response to high‐intensity auditory stimulation (94.5 dB). Thus, in the present study, we hypothesize replicating a similar physiological response to the highest auditory intensity (94.5 dBA), that is, an increase in EDA (phasic and tonic), a decrease in the initial seconds in HR, a decrease in PPG, and oxygenated hemoglobin. And for the new measurements supporting the hypothesis of an increase in sympathetic activity, we expect that the highest auditory intensity would produce: lower HRV values in the time domain, an increase in the LF/HF ratio in the HRV frequency domain, high power in the LFnu in PPG signal, and a lower PTT.

Therefore, this new study aims not only to replicate the results previously obtained to high‐intensity auditory stimulation (Muñoz‐Caracuel et al., 2021), but also to observe vasoconstriction due to sympathetic response at the cephalic and peripheral levels by adding to the analysis measures such as HRV, spectral analysis of PPG, and PTT.

## 2 MATERIAL AND METHODS

### 2.1 Ethical approval

The experiment followed the rules of the latest revision of the *Declaration of Helsinki* for human research (2013) and was approved by the portal de ética investigaciones biomédicas de la Junta de Andalucía.

### 2.2 Participants

Twenty‐five volunteer subjects (9 males and 16 females, mean ± age = 23.74 ± 2.89 years) participated in the study. As a selection criterion, all participants had normal hearing, and none of them had a history of neurological or psychiatric disorders. Before the study, they were informed about the procedures, and the experimental protocol and subsequently signed an informed consent form.

### 2.3 Procedure

**FIGURE 1:**
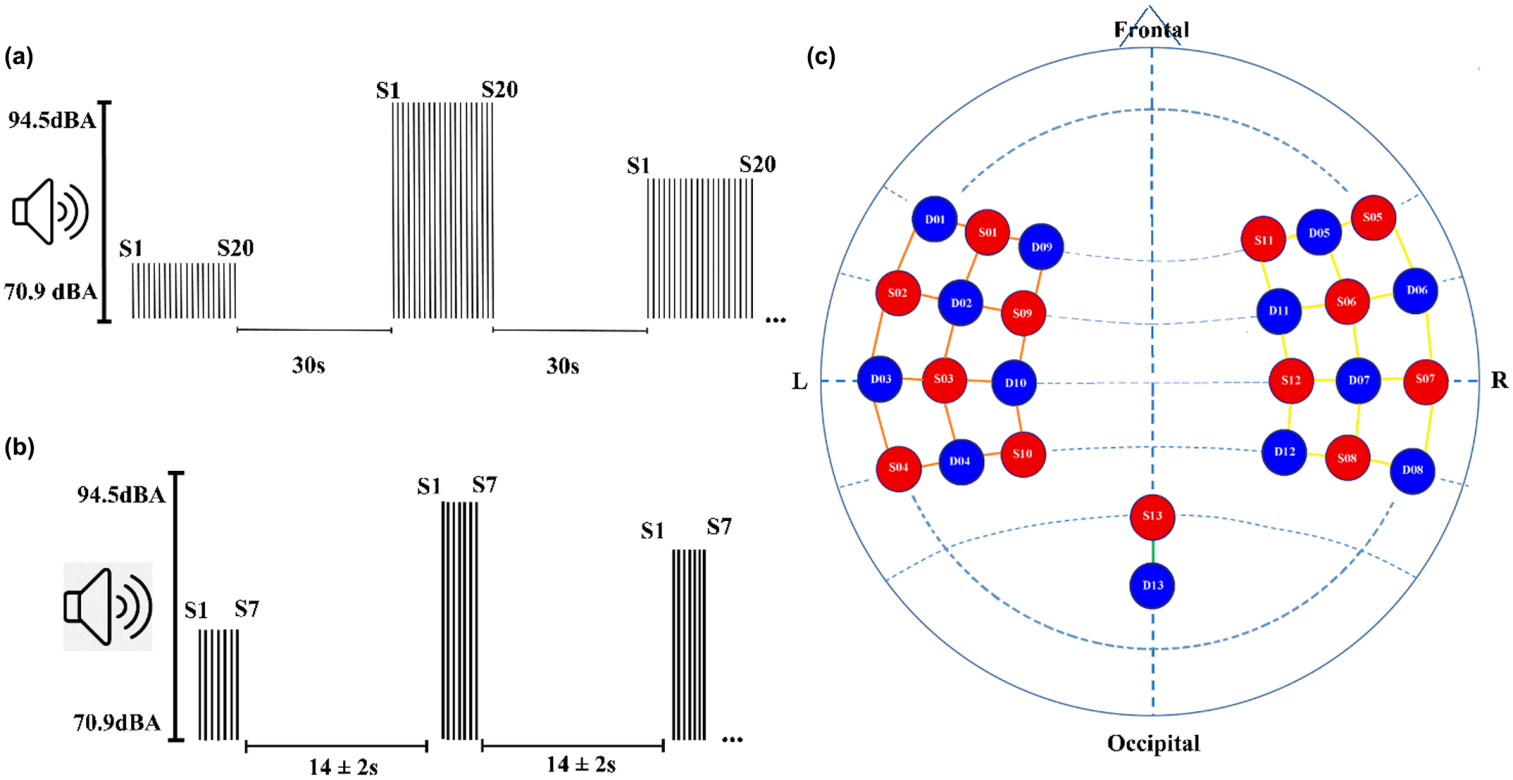
Experimental procedure. (a) Graphic of the stimulation process showing the 20 single stimuli (S1–S20) in each trial for each intensity and the 30‐s inter‐stimulus rest (b) graphic of the auditory stimulation protocol of Muñoz‐Caracuel et al. (2021) showing the 7 single stimuli (S1–S7) in each trial for each intensity and the rest period (14 ± 2 s) (c) fNIRS montage of the experiment showing the channels formed by the sources and detectors with a standard distance of 3 cm. The circles in red correspond to the location of light sources, the circles in blue to the light detectors. The lines in yellow correspond to the channels recording the right auditory cortex, in orange the left auditory cortex, and in green the visual channel.

Trials of five different tone intensities were presented (70.9, 77.9, 84.5, 89.5, 94.5 dBA) through two speakers connected to a computer using the E‐Prime 2.0 software package. Each trial included 20 tones of the same intensity, each with a duration of 70 ms with a frequency of 1003 Hz. (5 ms rise/fall time, sampled at 44.1 kHz) with 0.430 ms of interstimulus interval. The stimulus sequence was followed by 30 s of silence. The five auditory intensity trials were presented randomly to each experimental subject. In total, 35 trials were presented (seven trials for each intensity) as can be seen in Figure 1a. Initially, participants were instructed to move the less as possible, and to watch a silent movie, while the tones were played. The movie was employed as a distraction to increase tolerance and adherence to the experiment. No active responses were demanded. The data of the Muñoz‐Caracuel et al. (2021) (Hereinafter referred to as the Muñoz‐Caracuel experiment) study were also analyzed for the PTT and spectral PPG signal which was not previously analyzed. For the Muñoz‐Caracuel experiment, the same recording conditions as in the present report were used, but trials were formed only by seven consecutive stimuli and an intertrial interval of 14 ± 2 s. Twenty trials were delivered for each auditory intensity (Figure 1b). Methodological details of this experiment can be found in Muñoz‐Caracuel et al. (2021).

### 2.4 Signal acquisition and processing

#### 2.4.1 Peripheral signals

For the recording of the autonomic peripheral signals, an MP160 (BIOPAC Systems) with three different amplifier modules (PPG‐100C, EDA‐100C, and ECG‐100C) were used to obtain the PPG, EDA, and ECG signals, respectively. The pulse was recorded through a photoplethysmograph (TSD200 transducer with a wavelength of 860 ± 60 nm‐infrared light) placed on the index finger of the right hand. EDA was obtained using bipolar Ag‐AgCl finger electrodes placed on the index and middle fingers of the left hand. ECG was acquired using three Ag‐AgCl lead electrodes (positive, negative, and ground) placed on the left wrist, right wrist, and left ankle, respectively. The amplification gain was 100 in PPG, five in EDA, and 1000 in ECG, and the sampling frequency was 2000 Hz. Data acquisition was performed using AcqKnowledge v.5.0.1 software (BIOPAC Systems). Data filtering and processing were performed in Acknowledge v.5.0.1 (BIOPAC Systems), Matlab R2019b (MathWorks), Ledalab (Benedek & Kaernbach, 2010), HEPLAB (Perakakis, 2019), and KARDIA (Perakakis et al., 2010) software packages.

For PPG signal analysis in AcqKnowledge software, the raw signal was bandpass filtered from 0.2 to 5 Hz. Then, with a custom Matlab script, the time from −5 to 50 s was extracted for each intensity and trial, the absolute value was calculated by rectifying the signal and the 5 s baseline was subtracted. For the spectral analysis, the nonfiltered raw signal was analyzed in a customized Matlab script, where the 12‐s post‐stimulus were extracted, then spectral analysis was performed for each intensity and trial using the PLOMB Matlab function, and finally, the LF (0.04–0.15 Hz), and HF (0.15–0.4 Hz) values were normalized by the division by total power and multiplied by 100.

The nonfiltered EDA signal was analyzed by a custom Matlab script, in which the EDA signal was extracted between −5 and 50 s, and then was performed a baseline subtraction to obtain the changes in the event‐related SCL. A subsequent analysis of the EDA signal was performed with Ledalab software, using Continuous Decomposition Analysis (Benedek & Kaernbach, 2010) to decompose the recorded raw EDA signal into continuous phasic and tonic components. The parameters for the decomposition were optimized for each subject, following the software recommendations. This analysis allows us to obtain the values of the phasic measurements, having as input parameters: a time window of 1–5 s, and a minimum SCR amplitude of 0.01 μS, following the SCR analysis guidelines (Boucsein et al., 2012), (see Figure S3).

The ECG signal was filtered with a bandpass filter of 0.5–35 Hz (Ruha et al., 1997). this signal was then converted into HR, using the AcqKnowledge software. The HR signal was analyzed in a Matlab custom script in which the −5 to 50 s were extracted for each stimulus and trial, and the 5 s baseline was subtracted.

For the analysis of HRV, the ECG was taken unfiltered and the R peaks were found with the software HEPLAB using the PanTompkins function (Pan & Tompkins, 1985), the selection of R‐peaks was visually inspected for each subject to detect ectopic beats and to avoid misdetections. Then the inter‐beat‐intervals (IBIs) were exported to KARDIA software, for time and frequency domain analysis, and the spectral analysis was performed following the parameters of the software. First, the IBIs series were interpolated by cubic splines at a 2 Hz sample rate, subsequently was detrended by a constant, multiplied by a window Hanning function, and finally was calculated the discrete Fourier transform through the fast Fourier transform algorithm, in a 16 AR order. The frequency spectrum was divided into three bands: VLF (0–0.04 Hz), LF (0.04–0.15 Hz), and HF (0.15–0.5 Hz).

The ECG and PPG signals filtered were used to calculate PTT, this calculation was performed in the AcqKnowledge software, then with a custom Matlab script the window −5 to 50 s for stimuli and trial was extracted, the artifacts were rejected, and the baseline was subtracted.

Taking into account the low number of trials in the present experiment (7 trials), the data of the Muñoz‐Caracuel experiment, in which 20 trials were presented per auditory intensity, were used to replicate the PTT, and spectral PPG analysis, using the same procedure, described above. For all the peripheral recorded signals, motion artifacts were detected by ocular inspection and corrected using spline interpolation.

#### 2.4.2 Functional near‐infrared spectroscopy

The fNIRS signal was recorded with a NIRScoutXP (NIRx Medical Technologies) device with 13 LED sources and 13 detectors placed in temporal areas of both sides of the scalp and occipital lobe, obtaining 35 channels, as can be seen in the montage in Figure 1c. The fNIRS operate with two different wavelengths (760 and 850 nm) and avalanche photodiodes, which provide a high sensitivity to the optical signal. The source‐detector distance was 3 cm, a standard measure. The sampling rate was 4.8 Hz. In this montage, the short channels were not added since they were not available. However, considering that the purpose of the study was to measure at the head level the response at different intensity levels, and our previous study (Muñoz‐Caracuel et al., 2021) showed that in short channels a decrease in HbO also occurs at high stimulation, a visual channel was added to compensate this lack. The data acquisition was made with NIRStar 14.2 software (NIRx Medical Technologies).

For the signal processing, the fNIRS data were imported into Homer2 (Huppert et al., 2009). First, for pruning the noisy channels, the function enPruneChannels was performed, this algorithm removes channels with extreme values that saturated the signal (levels 0.03–2.5), or those channels that presented a high standard deviation (an SNR value of 5 was fixed to obtain a coefficient of variation of 17). For the reduction of the signal motion artifacts, the function hmrMotionCorrectionWavelet was applied, with an inter‐quartile range of 1.5. This function has proven to be quite robust in decreasing motion artifacts through wavelet decomposition (Brigadoi et al., 2014; Cooper et al., 2012; Molavi & Dumont, 2010). The signal was also bandpass filtered, between 0.01 and 0.5 Hz with the function *hmrBandpassFilt*.

Finally, for the remaining artifacts, the function hmrMotionArtifact (SDThresh = 15; AMPThresh = 0.7; tmotion = 1.0 and tmask = 1.0) was used, this function allows identifying persistent artifacts in the signal to be removed later, for a time window of −5 to 20 s around the stimulus. The hemoglobin concentrations were obtained employing the modified Beer–Lambert law with a differential partial pathlength factor of 6 for 760 nm and 5 for the 850 nm wavelength (Scholkmann et al., 2010). The filtered and processed signal was finally averaged over a window of −4 to 30 s for further statistical analysis, for each subject and each of the 5 stimulus intensities.

#### 2.4.3 EEG recording and analysis

To test that the 5 presented auditory intensities produced the expected central effects, the EEG was recorded in Cz electrode and averaged independently for each sound intensity. Figure S1 shows the auditory‐evoked potentials induced by the first stimulus in each stimulus train. The intensity‐dependent response of N1 is appreciated, as has been described previously (Adler & Adler, 1989; Muñoz‐Caracuel et al., 2021; Näätänen & Picton, 1987), Therefore, a brain‐dependent response increases with auditory intensity at the cerebral level is validated, and the possible changes in peripheral and central measures can be considered as a consequence of the brain processing of the different intensities of auditory stimuli. These data were not further analyzed, because it is beyond the scope of the present study.

### 2.5 Statistical analysis

#### 2.5.1 Peripheral signals

For the mean value of the absolute PPG and EDA signal (SCL), the time window of 4–8 and 2–8 s were selected, respectively, for the analysis of HR three time windows were selected 1–3, 3.5–5.5, and 6–8 s. The criterion for selecting these time windows was to use the same time windows as in our previous study for EDA, HR, and PPG (Muñoz‐Caracuel et al., 2021).

For the new signals recorded HRV, PTT, and spectral PPG, the criteria for selecting the time windows were the following: For HRV, in the time and frequency domains, the whole post‐stimulus time window (50 s) was used due to the requirements of the HRV analysis, which needs a large time window to obtain reliable measurement (Kuusela, 2012), for the spectral PPG, a time window of 12‐s post‐stimulus was selected for the data from both experiments to maintain the same time for the analysis, and for PTT a collapse of the five intensities and both experiments were performed to find the points of change in the signal, finding a signal decrease at the 6–8 and 8–10 s times (see Figure S2) both experiments and. Please notice that the latter approach is not biased given that is independent of auditory intensity conditions and the experiment.

For the HRV analysis in the frequency domain were considered the values of absolute LF (0.04–0.15 Hz), absolute HF (0.15–0.5 Hz), normalized LF (LFnu), normalized HF (HFnu), and the LF/HF ratio. For the time domain, the values of RMSSD, and SDNN were analyzed according to the values extracted from the Kardia software. Both within a time window of 50‐s post‐stimulus. For the spectral analysis of the PPG signal, the values of the normalized LFnu, HFnu, and the LF/HF ratio were considered for the analysis.

Finally, for phasic EDA, the values exported in Ledalab for the statistical analysis were as follows: AmpSum (SCR‐amplitudes of significant SCRs), nSCR (number of SCRs), SCR (average phasic driver in time window), PhasicMax (maximum value of phasic activity in the time window), and the value of the mean of tonic EDA activity, all the results were exported in absolute values and *z*‐scores.

For the statistical analysis, the data of the peripheral measurements were averaged independently for each intensity by subject, in the selected time windows, and a one‐way PERMANOVA with the auditory intensities as a factor was computed. For signals with two‐time windows such as PTT and HR, a two‐way PERMANOVA was performed. When PERMANOVA was significant, *t*‐tests with a false discovery rate correction (FDR) were applied for each factor. For the PERMANOVA the software PAST (Hammer et al., 2001) was employed. PERMANOVA uses a permutation test and does not have the requirements of normality of ANOVA.

#### 2.5.2 Functional near‐infrared spectroscopy

For the fNIRS analysis, regions of interest (ROIS) were defined using the software FOLD (Zimeo Morais et al., 2018), which allows defining the scalp locations in the regions of interest, for this study: left and right auditory cortex (RAC), and the visual cortex. It is important to note, that by not performing emitters and sensors head locations measurements and/or structural MRI studies on the participants, it is unsuitable to claim that the standardized regions of the software FOLD display the activity of the ROIS, so we preferred to use a larger number of channels to allow less error bias than if concentrating analysis in the channel proposed as the primary auditory cortex. The hemodynamic responses for each channel configuring each of the three ROIS analyzed were collapsed. For the selection of the time window for the analysis, since the auditory stimulation time was approximately 20 s in the present study, the first‐time window was selected from 3 to 12 s (including the both previously studied windows 3–8 and 8–12 s in Muñoz‐Caracuel et al., 2021), and adding a new one from 13 to 23 s to cover the total stimulation time.

For the statistical analysis a two‐way PERMANOVA with the factor auditory intensities (70.9, 77.9, 84.5, 89.5, and 94.5 dBA), and ROIS (left auditory cortex [LAC], RAC, visual cortex), was performed. This analysis was computed by each time window (3–12 and 13–23 s, after stimulus). The post hoc comparisons were computed with FDR correction.

Finally, to eliminate the common head global signal (both peripheral scalp and cerebral) from the auditory cortex channels, a regression of the visual channel activity vs the auditory cortex channels was performed. The reason for performing the regression with the visual channel is that in the present report short channels were not recorded, and this procedure could minimize the contribution of the scalp and global activity brain contribution to the fNIRS signal recorded in temporal sites. The procedure for the regression was to regress the visual channel activity versus auditory cortex activity and subtract the regressed signal from the fNIRS activity in the LAC and RAC. For the statistical analysis a two‐way PERMANOVA with the factor auditory intensities (70.9, 77.9, 84.5, 89.5, and 94.5 dBA), and ROIS (LAC and RAC), was performed. This analysis was computed by each time window (3–12 s and 13–23 s, after stimulus). Lastly, a *t*‐test compared to the baseline (FDR corrected) was performed between the fNIRS signal at different intensities. Table 1 includes all the metrics analyzed, their possible interaction, and meaning in response to stimulation.

**TABLE 1:** Metrics and meaning of the analyzed signals

Type of responses	Signals	Metrics	Meaning
Vascular responses	PPG	Mean absolute PPG	The PPG signal has been related to changes in blood volume or blood volume pulse. The decrease in PPG could be related to a decrease in blood volume and vasoconstriction (Abay & Kyriacou, 2022; Grote et al., 2003)
	PSD PPG	LFnu HFnu LF/HF	High power in the Low‐frequency fluctuations of PPG has been proposed as a sensitive indicator of sympathetic control of arteriolar circulation and has also been associated with the regulation of peripheral vascular tone (Bernardi et al., 1996; Karavaev et al., 2021)
	PTT	Mean PTT	This measure is used to assess sympathetic activity in the face of an environmental change or stressful condition. It has been proposed that the decrease in PTT is generated by a vasoconstriction response (Budidha & Kyriacou, 2014; Mol et al., 2020)
Electrodermal responses	SCL	Mean EDA Tonic EDA	SCL represents the slowly varying baseline level of the SC. Variations in SCL are believed to reflect slow changes in SNA dynamics (Greco et al., 2016)
	SCR	Ampsum nSCR SCR PhasicMax	SCR reflects the short‐time response to the stimulus (Greco et al., 2016)
Cardiac responses	HR	Mean HR	HR represents the heart's modulation of beats, which in turn are innervated by the ANS, and its acceleration or deceleration represents changes in response to external or stressful stimuli (Graham & Clifton, 1966; Sokolov, 1963; Vila et al., 2007)
	HRV (Time domain)	RMSSD SDNN	The RMSSD and SDNN reflect the variations of the R‐R mainly for the high‐frequency (HF) component and have been associated with the vagal tone (Minarini, 2020)
	HRV (frequency domain)	aLF aHF LFnu HFnu LF/HF	HRV is associated with health and self‐regulatory capacity, and adaptability (Minarini, 2020) An increase in LF power is assumed to indicate an increase in sympathetic activity, and a higher value of the LF/HF ratio indicates sympathetic predominance. But these assumptions are still under discussion (Ernst, 2014, Kiyono et al., 2017, Reyes del Paso et al., 2013)
Cerebral responses (peripheral and central)	fNIRS	HbO HbR HbT	fNIRS is based on the assumption that active neurons induce a hemodynamic response in the cerebral vascular system that produces an increase in HbO and a decrease in HbR concentrations. Thus, this method analyzes the cortical activation of different brain regions (Pinti et al., 2020)

Abbreviations: EDA, electrodermal activity; fNIRS, functional near‐infrared spectroscopy; HbO, hemoglobin in the brain for oxygenated; HbR, hemoglobin in the brain for deoxygenated; HbT, total hemoglobin; HFnu, normalized HF; HR, heart rate; HRV, heart rate variability; LFnu, normalized LF; PPG, photoplethysmography; PSD, power spectral density; PTT, pulse transit time; RMSSD, root mean square of successive RR interval differences; SCL, skin conductance level; SCR, skin conductance response.

## 3 RESULTS

### 3.1 Peripheral signals

**FIGURE 2:**
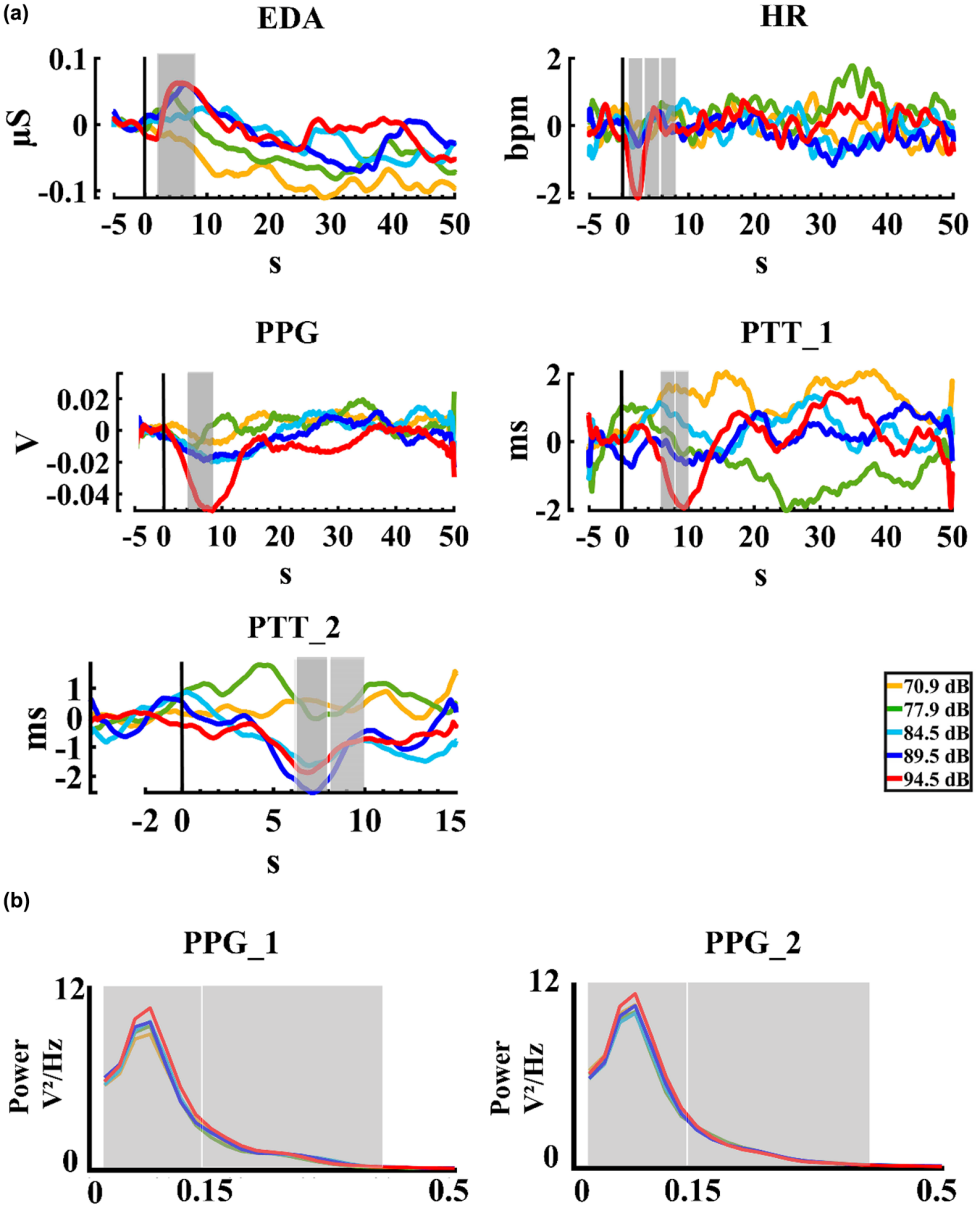
Peripheral signals. (a) Amplitude changes for: electrodermal activity (EDA), heart rate (HR), absolute peripheral pulse (PPG), pulse transit time (PTT_1) in the five auditory intensities (70.9, 77.9, 84.5, 89.5, and 94.5 dBA), and PTT_2 for Muñoz‐Caracuel et al. (2021) PTT data. (b) Normalized power spectral density of the PPG signal for present (PPG_1), and Muñoz‐Caracuel (PPG_2) experiments. The selected windows for the statistical analysis are labeled in gray.

The responses of the peripheral signals for the five auditory intensities in the present experiment are shown in Figure 2a, and the statistical results for the measurements are displayed in Table 2, and Table 3 for the Muñoz‐Caracuel experiment. For absolute PPG in the figure of the amplitude (Figure 2a) a decrease of the signal for the highest intensity can be observed. Accordingly, in the statistical results for the metric mean absolute PPG, the PERMANOVA showed an effect of intensity. The *t*‐tests (FDR corrected) presented a significant difference between the intensities 1 (70.9 dBA), 2 (77.9 dBA), 3 (84.5 dBA), and 4 (89.5 dBA) with the intensity 5 (94.5 dBA), showing a signal decrease (*p* = 0.007, Int5 < Int1; *p* = 0.024, Int5 < Int2; *p* = 0.018; Int5 < Int3, *p* = 0.043; Int5 < Int4).

**TABLE 2:** PERMANOVA results for peripheral measurements for the present experiment

Type of responses	Measure	Metrics	Factor	*p*‐value PERMANOVA	*η* ^2^
Vascular responses	PPG	Mean absolute PPG	**Intensity**	**0.006**	**0.135**
	PSD PPG	LFnu	**Intensity**	**0.057**	**0.094**
		HFnu	Intensity	0.155	0.068
		LF/HF	**Intensity**	**0.056**	**0.093**
	PTT	Mean PTT	**Intensity**	**0.002**	**0.084**
			Time window	0.458	0.062
			Interaction	0.780	0.087
Electrodermal responses	SCL	Mean EDA	Intensity	0.292	0.052
		Tonic EDA	Intensity	0.734	0.021
	SCR	Ampsum	Intensity	0.174	0.066
		nSCR	Intensity	0.309	0.050
		SCR	Intensity	0.235	0.058
		PhasicMax	Intensity	0.405	0.042
Cardiac responses	HR	Mean HR	Intensity	0.258	0.035
			**Time window**	**0.006**	**0.311**
			Interaction	0.467	0.061
	HRV (time domain)	RMSSD	**Intensity**	**0.029**	**0.092**
		SDNN	Intensity	0.289	0.052
	HRV (frequency domain)	aLF	Intensity	0.913	0.013
		aHF	**Intensity**	**0.084**	**0.066**
		LFnu	Intensity	0.316	0.049
		HFnu	Intensity	0.323	0.049
		LF/HF	Intensity	0.268	0.055

Abbreviations: EDA, electrodermal activity; HFnu, normalized HF; HR, heart rate; HRV, heart rate variability; LFnu, normalized LF; PPG, photoplethysmography; PSD, power spectral density; PTT, pulse transit time; RMSSD, root mean square of successive RR interval differences; SCL, skin conductance level; SCR, skin conductance response. Bold type is used in the table to highlight significant results.

**TABLE 3:** PERMANOVA results for vascular measurements for Muñoz‐Caracuel experiment

Measure	Metrics	Factor	*p*‐value PERMANOVA	*η* ^2^
PSD PPG_2	LFnu	**Intensity**	**0.001**	**0.146**
	HFnu	Intensity	0.562	0.024
	LF/HF	**Intensity**	**0.027**	**0.086**
PTT_2	Mean PTT	**Intensity**	**0.008**	**0.073**
		Time window	0.188	0.093
		Interaction	0.779	0.047

Abbreviations: HFnu, normalized HF; LFnu, normalized LF; PPG, photoplethysmography; PSD, power spectral density; PTT, pulse transit time. Bold type is used in the table to highlight significant results.

For the PPG spectral analysis of the present experiment data (Figure 2b), the HFnu power was not significant, while the LFnu power, and LF/HF ratio showed a statistical trend. The post hoc analysis FDR corrected showed for the LFnu a significant difference between the intensity 1 (70.90 dBA) with the intensity 5 (94.5 dBA) (*p* = 0.016, Int5 > Int1), and similarly in the LF/HF ratio a difference between the intensity 1 (70.9 dBA) with the intensity 5 (94.5 dBA) (*p* = 0.048, Int5 > Int1), was found. Likewise, in the Muñoz‐Caracuel experiment, the PERMANOVA showed an effect of intensity in the LFnu power, and in the LF/HF ratio, but the HFnu power did not show significant results. The post hoc analysis for this data with the FDR correction showed for the LFnu a significant difference between the intensity 2 (77.9 dBA) and 3 (84.5 dBA) with the intensity 5 (94.5 dBA) (*p* = 0.009, Int5 > Int2, *p* = 0.007, Int5 > Int3), and for the LF/HF ratio not differences were found.

**FIGURE 3:**
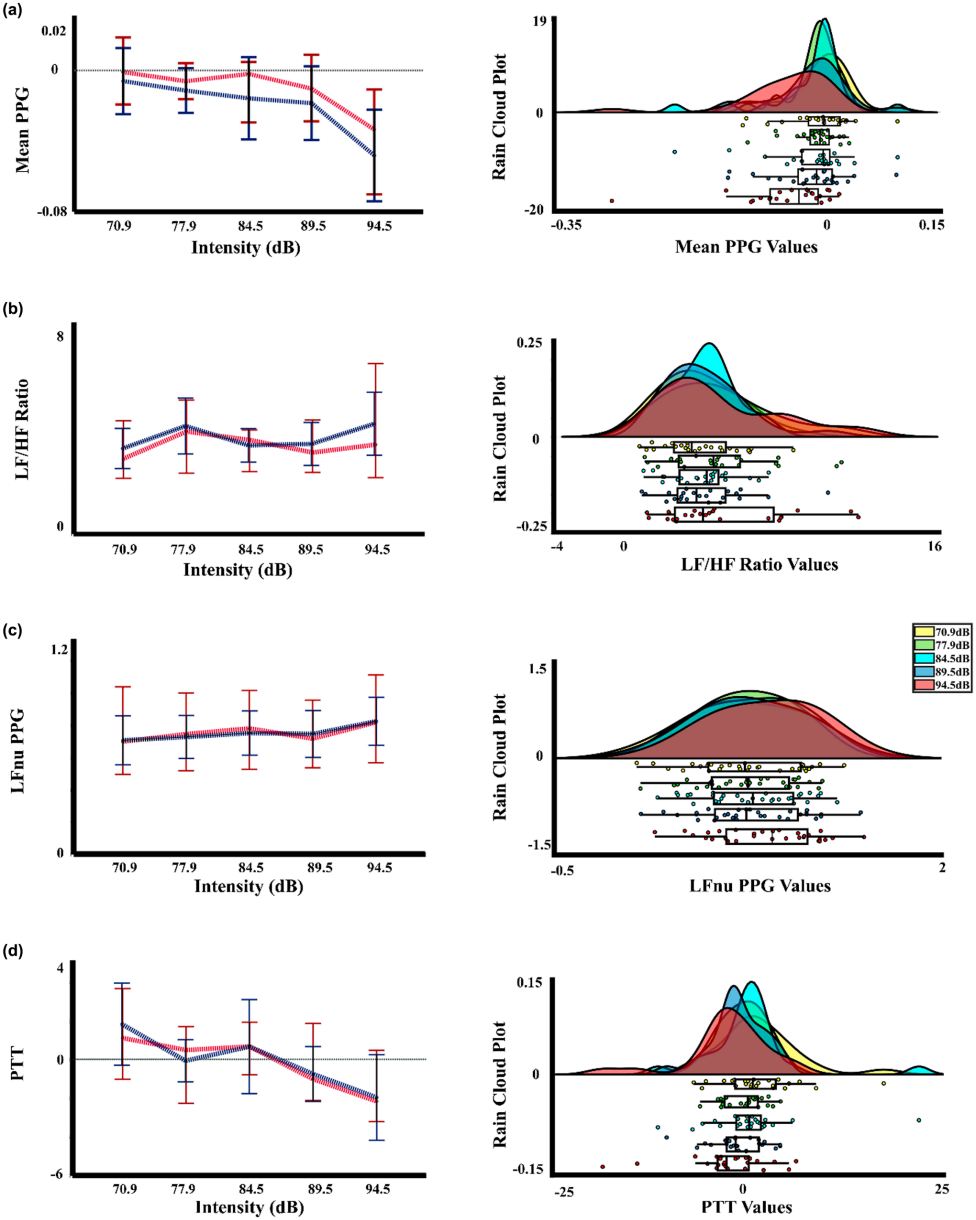
Median, mean values, and a rain cloud plot for the median values of the significant peripheral vascular measurements in the present experiment. (a) Absolute PPG signal by intensity. (b) LF/HF ratio for the PSD of the PPG signal by intensity. (c) LFnu for the PSD of the PPG signal by intensity. (d) PTT by intensity. Median: red, mean: blue. PPG, photoplethysmography; PSD, power spectral density; PTT, pulse transit time.

**FIGURE 4:**
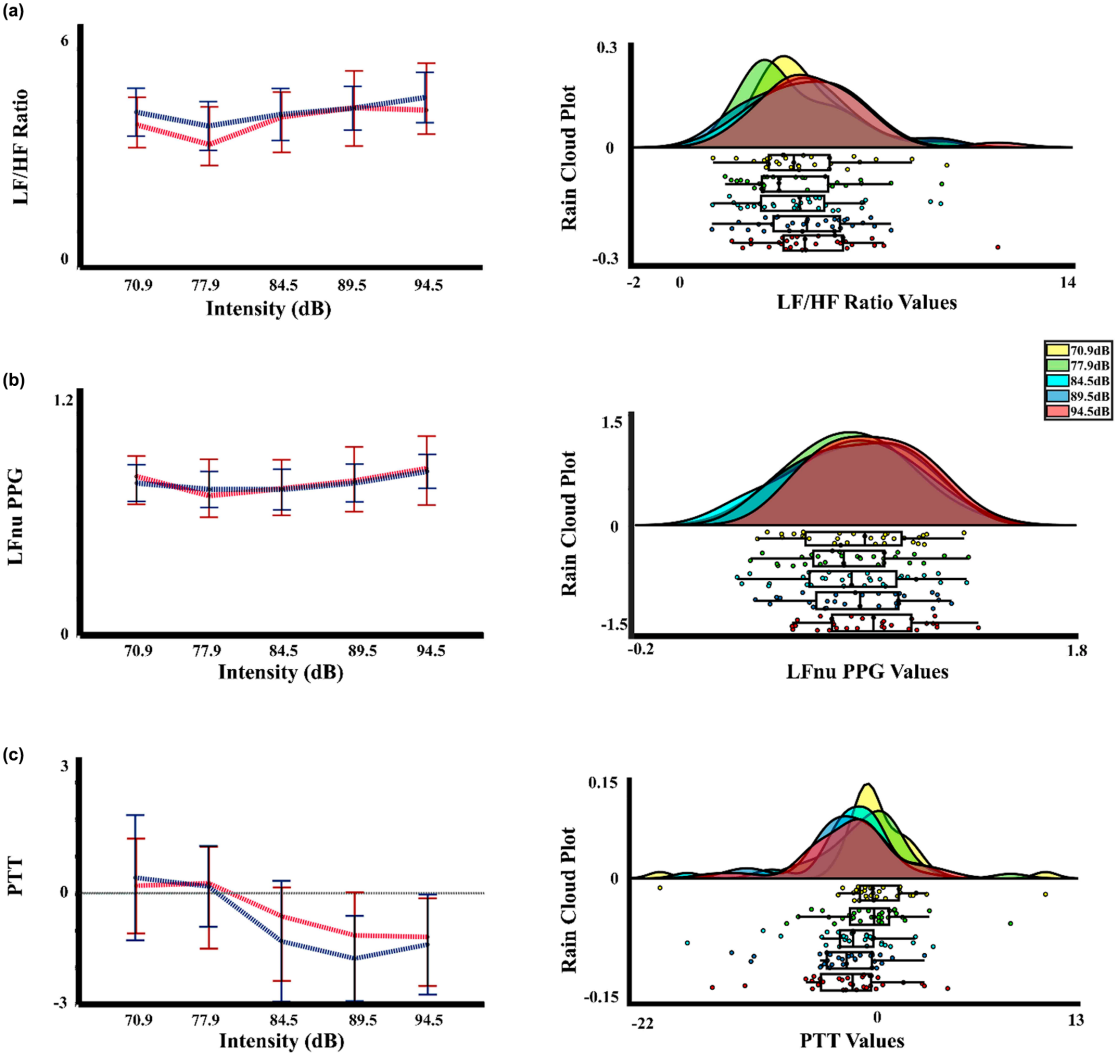
Median, mean values, and a rain cloud plot for the median values of the significant peripheral vascular measurements for Muñoz‐Caracuel data. (a) LF/HF ratio for the PSD of the PPG signal by intensity. (b) LFnu for the PSD of the PPG signal by intensity. (c) PTT by intensity. Median: red, mean: blue. PPG, photoplethysmography; PSD, power spectral density; PTT, pulse transit time.

Following the vascular measures, for the PTT of the present experiment, the two‐way PERMANOVA showed an effect of intensity. The post hoc not corrected showed differences between intensity 1 (70.9 dBA) with the intensity 4 (89.5 dBA) (*p* = 0.047; Int4 < Int1). However, the FDR correction did not show any significant differences between the intensities. In the same way, for the Muñoz‐Caracuel experiment data, the two‐way PERMANOVA of the PTT signal showed an effect of intensity. The *t*‐test not corrected found significant differences between the intensity 2 (77.9 dBA) with the intensity 4 (89.5 dBA) and 5 (94.5 dBA) (*p* = 0.013, Int4 < Int2; *p* = 0.047, Int5 < Int2). However, the FDR correction removes the significant differences. Figures 3 and 4 display the mean and median, and rain cloud plots for the vascular measurements that obtained significant effects in the PERMANOVA for the present experiment and Muñoz‐Caracuel, showing both a clear signal decrease for the 94.5 dBA intensity in PTT and PPG, but an increase in the same intensity in the LFnu power of PPG.

For the EDA responses, although in Figure 2a, an amplitude increase for auditory intensities of 89.5 and 94.5 dBA is noticeable around 2‐ to 8‐s post‐stimulus, the PERMANOVA for the Mean EDA shows nonsignificant results in the analysis performed with a custom script. Neither, did it show significant differences in the EDA phasic measures extracted from Ledalab (Ampsum, nSCR, SCR, PhasicMax), nor tonic EDA.

**FIGURE 5:**
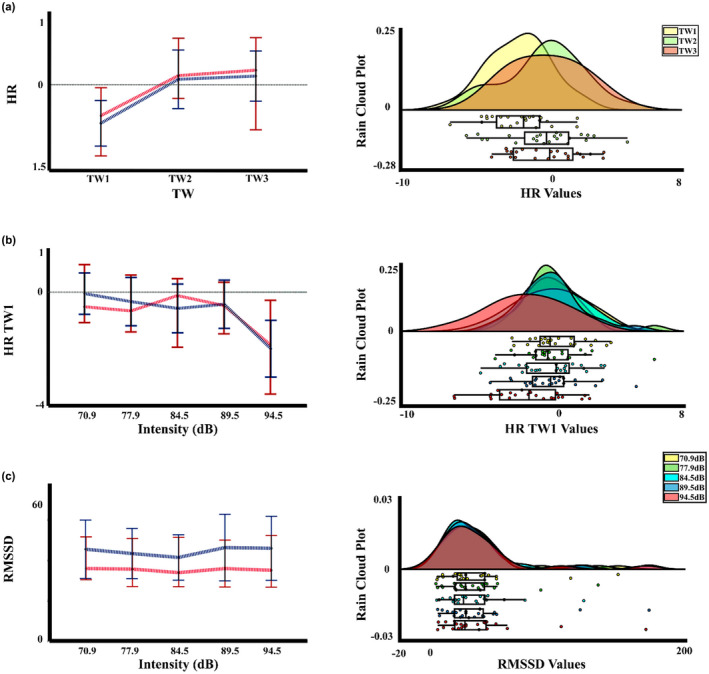
Median, mean values, and a rain cloud plot for the median values of the significant peripheral cardiac measurements. (a) Heart rate (HR) signal for the effect of time window. (b) HR of the first time window by intensity. (c) Root mean square of successive differences (RMSSD) by intensity. Median: red, mean: blue.

In the cardiac response, for the HR signal, the two‐way PERMANOVA showed an effect for the time window. When *t*‐tests (FDR corrected) were computed, significant differences between the time window 1 with the time windows 2 and 3 were found (*p* > 0.001, TW1 < TW2; *p* > 0.001, TW1 < TW3), showing a decrease in the signal in the firsts 3 s. No interaction of the effects of time window by intensity was found. However, given that Figure 2a, suggested that differences in HR due to auditory intensity in the time window 1, *t*‐tests (FDR corrected) for the first time window were computed, and significant differences between intensity 1 (70.9 dBA) with the intensity 5 (94.5 dBA) (*p* = 0.031, Int5 < Int1) were obtained. For HRV measures the PERMANOVA showed an effect of intensity for the metric RMSSD, the uncorrected comparisons showed a difference between intensities 1 (70.9 dBA) and 3 (84.5 dBA) (*p* = 0.033, Int3 < Int1), however when the FDR correction is performed there is not differences between the intensities. Likewise, the PERMANOVA showed a statistical trend of the effect intensity for the aHF of HRV, but the comparisons did not show any significant differences between the intensities. Figure 5 displays the mean and median, and rain cloud plots for the cardiac measures that obtained significant effects in the PERMANOVA.

### 3.2 Functional near‐infrared spectroscopy

**FIGURE 6:**
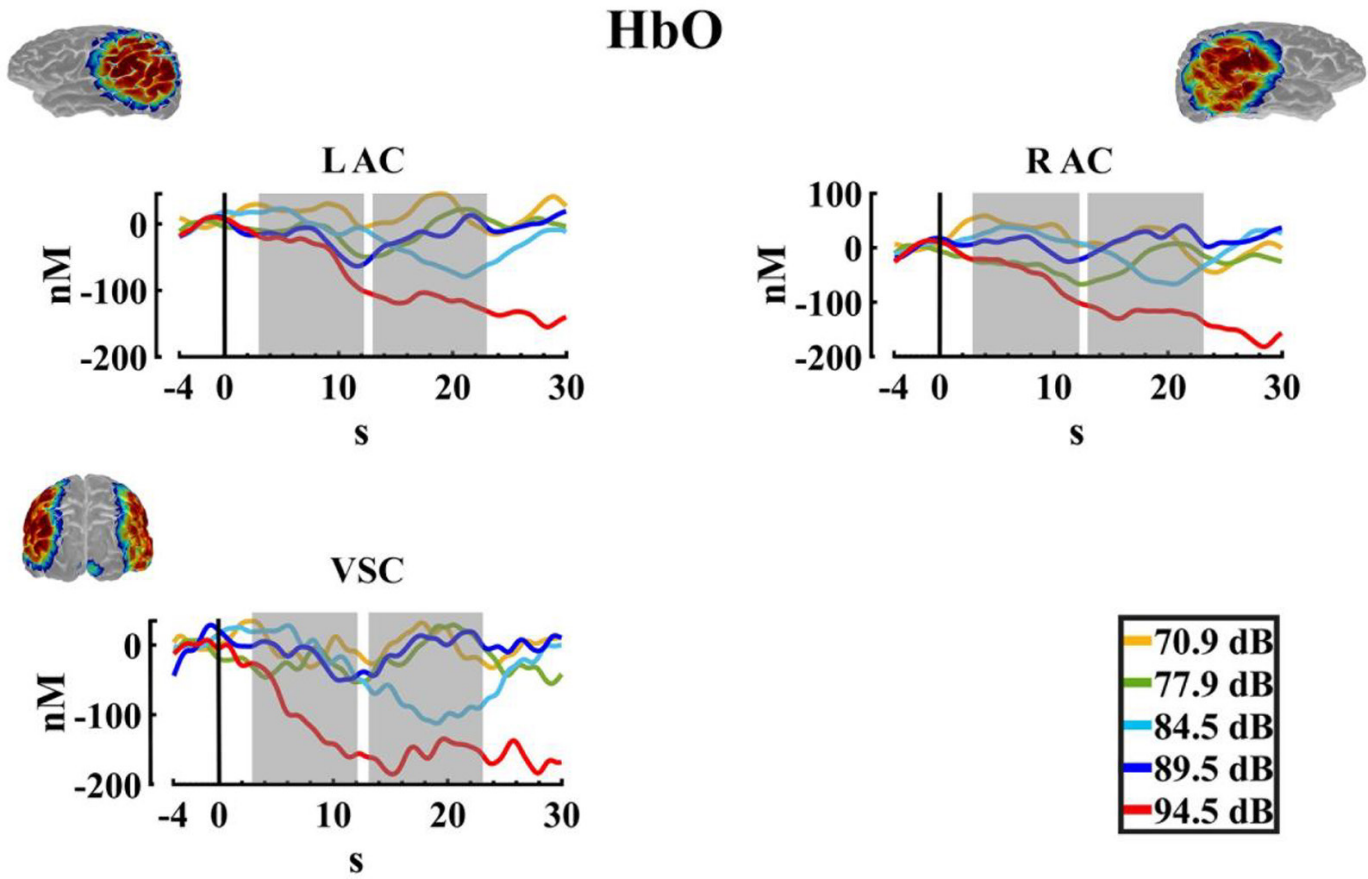
Oxyhemoglobin changes. Average change in the oxyhemoglobin (ΔHbO) for the five auditory intensities (70.9, 77.9, 84.5, 89.5, and 94.5 dBA) in the three regions of interest (LAC, left auditory cortex; RAC, right auditory cortex; VSC, visual cortex). The selected windows for the statistical analysis are marked in gray. The sensitivity maps obtained with the fNIRS montage, as indicated by AtlasViewer software, are inserted.

**FIGURE 7:**
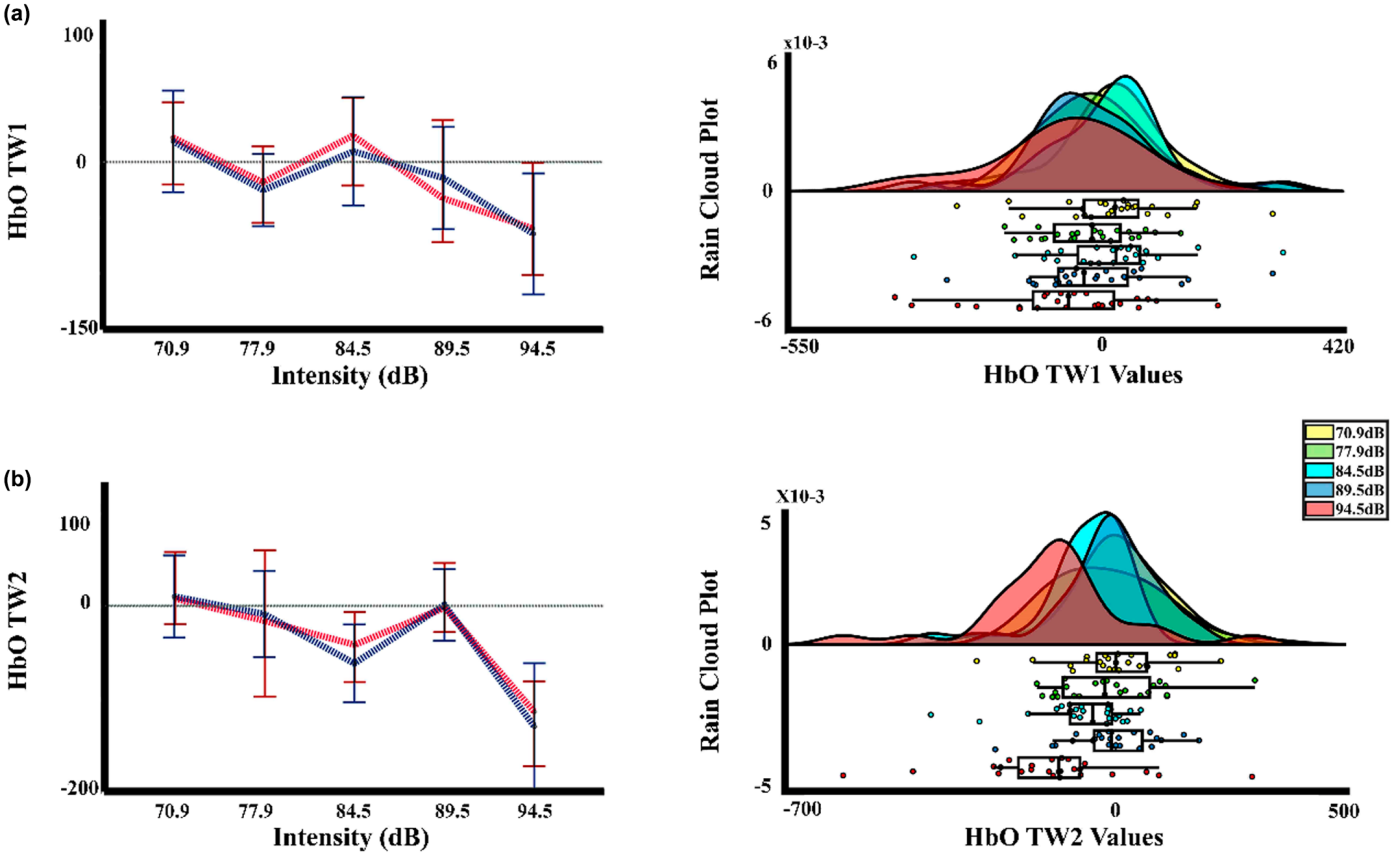
Median, mean values, and a rain cloud plot of the median values for the significant oxyhemoglobin results. (a) ΔHbO for the first time window, with the collapse of the ROIS, for the five auditory stimuli, and (b) ΔHbO in the second time window, with the collapse of the ROIS, for the five auditory stimuli. Median: red, mean: blue.

The results for the cerebral responses (Peripheral and central) are shown in Table 4. For HbO, Figure 6 shows a pronounced decrease in the amplitude of the oxyhemoglobin in the higher intensity, that is, 94.5 dBA, which seems to be generalized to all ROIS. Accordingly, the two‐way PERMANOVA (ROIS × auditory intensities) for both time windows showed an effect of auditory intensity. The post hoc analysis (FDR corrected) for the auditory intensity in the ΔHbO for the TW1, showed significant differences between intensity 1 (70.9 dBA) with the intensity 5 (94.5 dBA), showing a decrease in the concentrations in the higher auditory intensity (*p* = 0.035, Int5 < Int1), for the TW2 the *t*‐test FDR corrected indicated significant differences between the intensities 1 (70.9 dBA), 2 (77.9 dBA), 3 (84.5 dBA), 4 (89.5 dBA) with the intensity 5 (94.5 dBA) (*p* = 0.001, Int5 < Int1; *p* = 0.004, Int5 < Int2; *p* = 0.041; Int5 < Int3, *p* = 0.009; Int5 < Int4), and also a difference of the intensities 1 (70.9 dBA), and 4 (89.5 dBA) with the intensity 3 (84.5 dBA). (*p* = 0.047, Int3 < Int1; *p* = 0.046, Int3 < Int4). Figure 7 shows the median, mean, and rain cloud plot for the significant factors in the ΔHbO and the decrease at the highest intensity.

**TABLE 4:** PERMANOVA results for cerebral responses (peripheral and central)

Measure	Metrics	TW	Factor	*p*‐value PERMANOVA	*η* ^2^
fNIRS (not regressed with fNIRS of the visual channel)	HbO	1	**Intensity**	**>0.001**	**0.084**
			ROI	0.171	0.202
			Interaction	0.818	0.097
		2	**Intensity**	**>0.001**	**0.220**
			ROI	0.560	0.057
			Interaction	0.893	0.054
	HbR	1	Intensity	0.201	0.028
			**ROI**	**0.046**	**0.204**
			Interaction	0.283	0.099
		2	**Intensity**	**0.009**	**0.061**
			**ROI**	**0.047**	**0.301**
			**Interaction**	**0.031**	**0.125**
	HbT	1	**Intensity**	**0.002**	**0.065**
			ROI	0.554	0.064
			Interaction	0.930	0.059
		2	**Intensity**	**>0.001**	**0.201**
			ROI	0.976	0.002
			Interaction	0.972	0.035
fNIRS (regressed with fNIRS of the visual channel)	HbO	1	Intensity	0.401	0.028
			ROI	0.649	0.011
			Interaction	0.443	0.077
		2	Intensity	0.182	0.035
			ROI	0.677	0.023
			Interaction	0.956	0.016
	HbR	1	**Intensity**	**0.068**	**0.052**
			ROI	0.518	0.033
			Interaction	0.745	0.048
		2	Intensity	0.247	0.029
			ROI	0.437	0.111
			Interaction	0.808	0.053
	HbT	1	Intensity	0.227	0.039
			ROI	0.816	0.003
			Interaction	0.575	0.078
		2	**Intensity**	**0.004**	**0.086**
			ROI	0.930	0.001
			Interaction	0.961	0.018

Abbreviations: fNIRS, functional near‐infrared spectroscopy; HbO, hemoglobin in the brain for oxygenated; HbR, hemoglobin in the brain for deoxygenated; HbT, total hemoglobin. Bold type is used in the table to highlight significant results.

**FIGURE 8:**
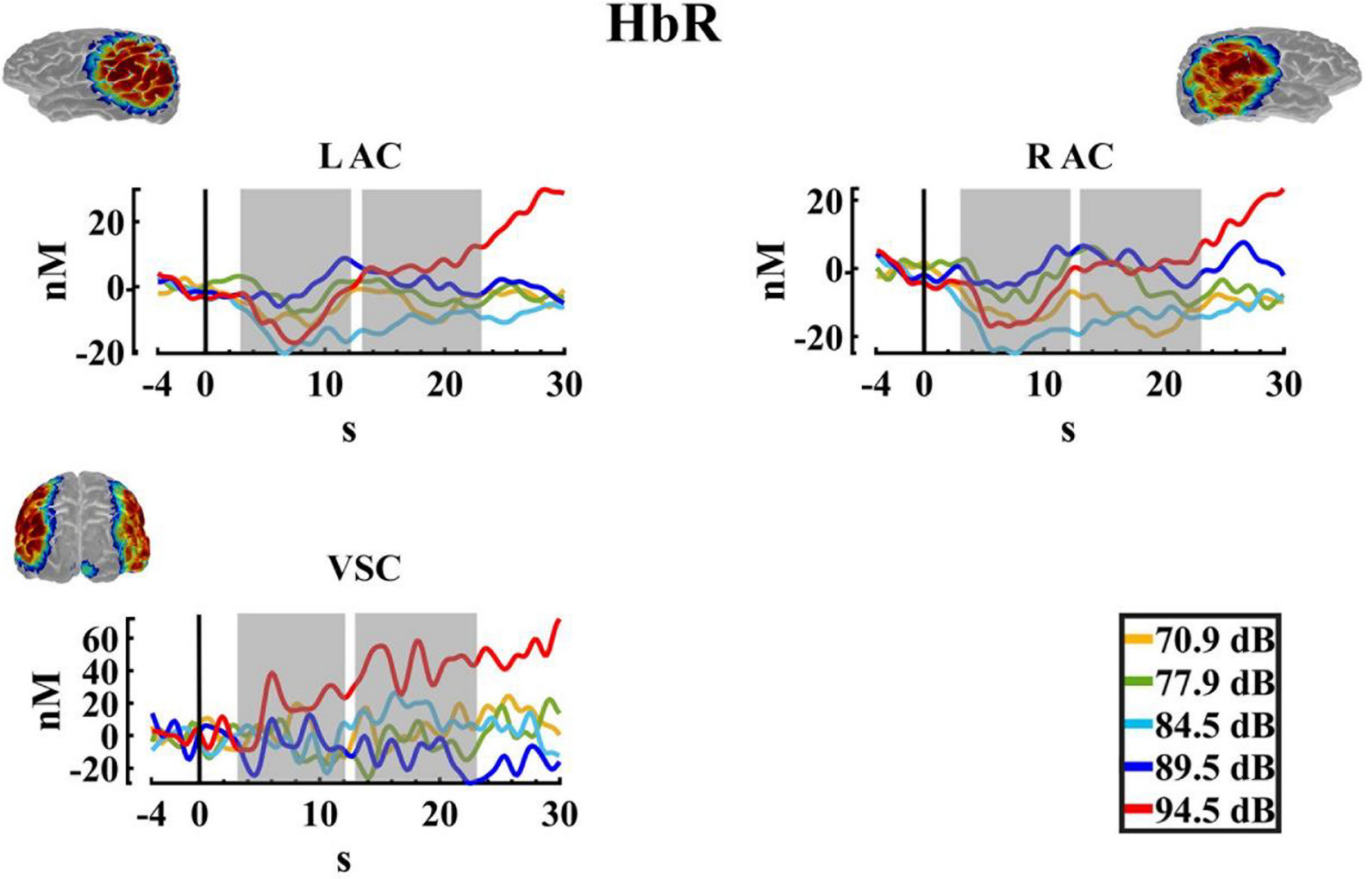
Deoxyhemoglobin changes. Average change in the deoxyhemoglobin (ΔHbR) for the five auditory intensities (70.9, 77.9, 84.5, 89.5, and 94.5 dBA) in the three regions of interest (LAC, left auditory cortex; RAC, right auditory cortex; VSC, visual cortex). The selected windows for the statistical analysis are marked in gray. The sensitivity maps obtained with the fNIRS montage, as indicated by AtlasViewer software, are inserted.

**FIGURE 9:**
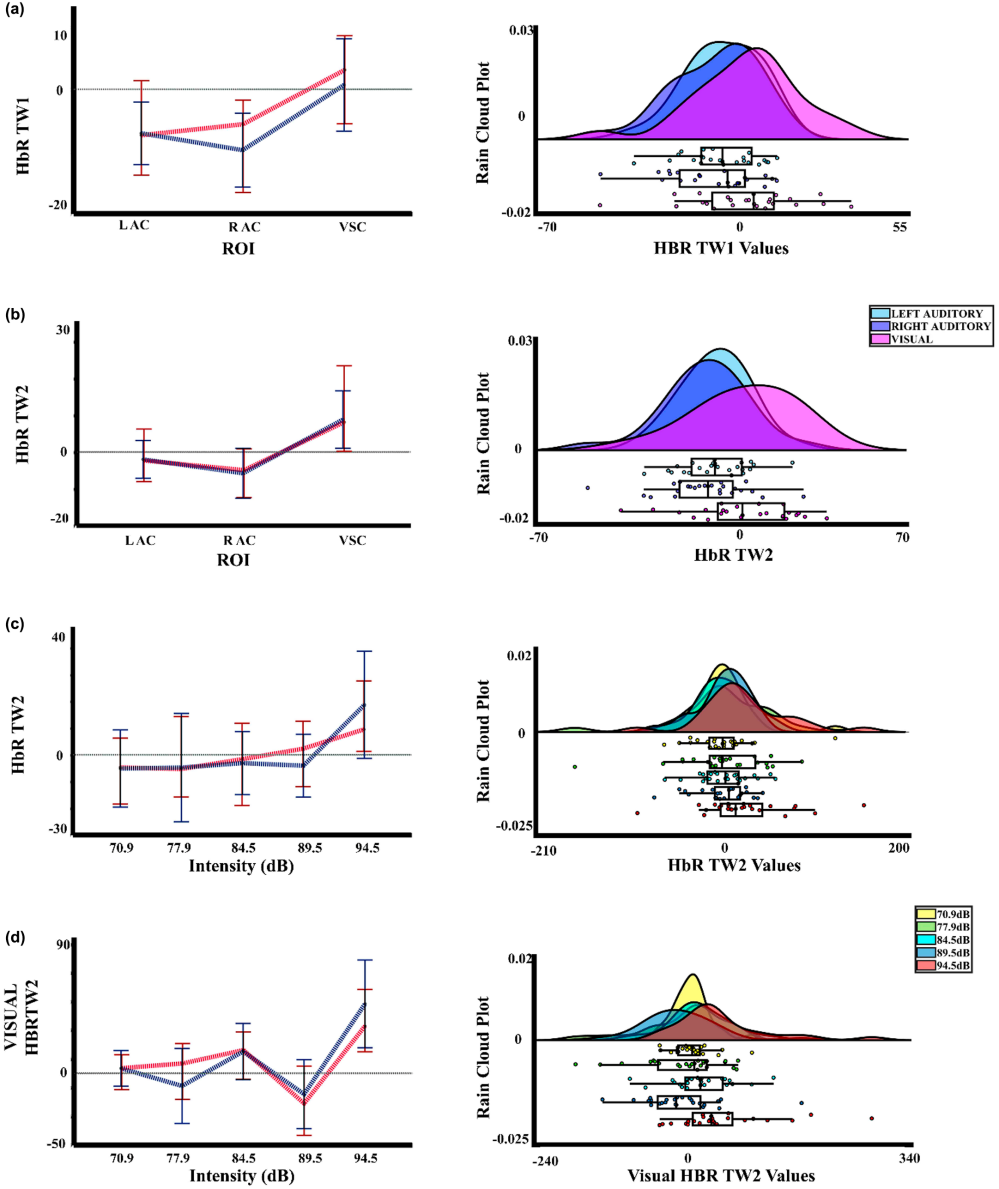
Median, mean values, and a rain cloud plot of the median values for the significant deoxyhemoglobin results. (a) ΔHbR signal in the first time window with the five auditory intensities collapsed, for the three ROIS. (b) ΔHbR signal in the second time window with the five auditory intensities collapsed, for the three ROIS. (c) ΔHbR in the second time window, with the collapse of the ROIS, for the five auditory stimuli. (d) ΔHbR of the five auditory stimuli in the visual channel. Median: red, mean: blue.

Figure 8 shows an increase in the deoxyhemoglobin concentration for the higher intensity, especially for the second time window. In the statistical analysis, for the ΔHbR the two‐way PERMANOVA (ROIS × auditory intensities) for the TW1 showed an effect of ROIS. The *t*‐test comparisons (FDR corrected) showed significant differences between the visual cortex and the RAC (*p* = 0.023; RAC < visual cortex). On the other hand, the PERMANOVA for the TW2 showed an effect for the intensity, ROI, and interaction. The post hoc for the intensity effect in this time window showed differences between the intensity 1 (70.9 dBA), 3 (84.5 dBA) and 4 (89.5 dBA), with the intensity 5 (94.5 dBA) (*p* = 0.012, Int5 > Int1; *p* = 0.036, Int5 > Int3; *p* = 0.044, Int5 > Int4), but the FDR correction did not show any significant difference. For the ROI effect, significant differences between the visual cortex with both auditory cortexes were found with the FDR corrected comparisons (*p* = 0.005; RAC < visual cortex, *p* = 0.012; left auditory cortex < visual cortex). And finally, for the interaction between ROI and intensity, the post hoc analysis FDR corrected just showed significant differences between the intensities in the visual cortex, with higher values for the intensity 5 (94.5 dBA) in comparison with the intensities 1 (70.9 dBA), 3 (84.5 dBA), and 4 (89.5 dBA) (*p* = 0.014, Int5 > Int1; *p* = 0.030, Int5 > Int3; *p* = 0.017, Int5 > Int4). Figure 9 displays the median, mean, and rain cloud plot, for the ΔHbR significant factor (ROIS), displaying a difference between both auditory areas and the visual cortex, and a higher amplitude for the highest intensity in this chromophore.

**FIGURE 10:**
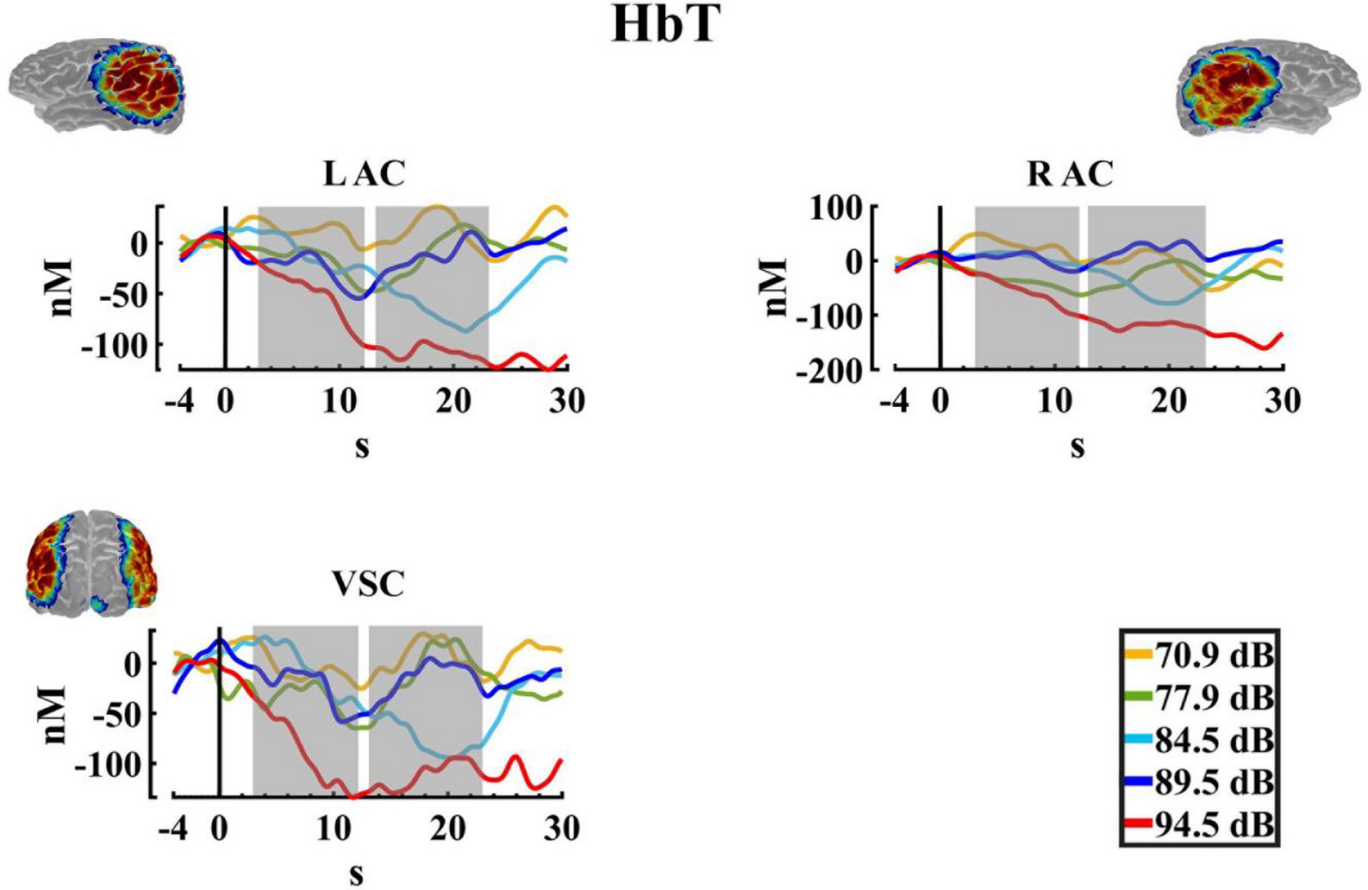
Total hemoglobin changes. Average change in the total hemoglobin (ΔHbT) for the five auditory intensities (70.9, 77.9, 84.5, 89.5, and 94.5 dBA) in the three regions of interest (LAC, left auditory cortex; RAC, right auditory cortex; VSC, visual cortex). The selected windows for the statistical analysis are marked in gray. The sensitivity maps obtained with the fNIRS montage, as indicated by AtlasViewer software, are inserted.

**FIGURE 11:**
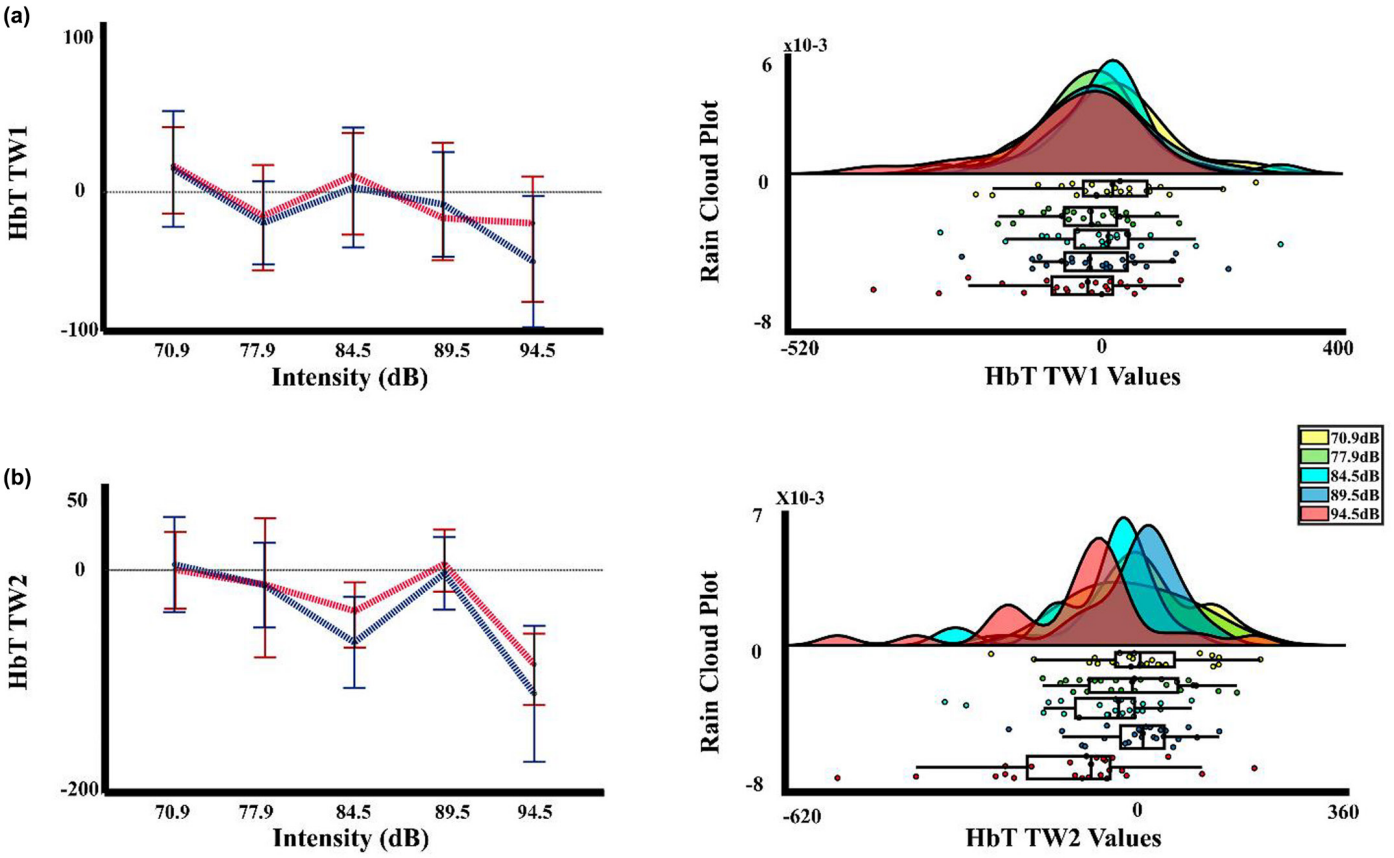
Median, mean values, and a rain cloud plot of the median values for the significant total hemoglobin results. (a) ΔHbT in the first time windows with the collapse of the ROIS, for the five auditory stimuli, and (b) ΔHbT in the second time window, with the ROIS collapsed, for the five auditory stimuli. Median: red, mean: blue.

For the ΔHbT, Figure 10 shows the response of this chromophore being similar to the ΔHbO, with a decrease for the highest intensity. The two‐way PERMANOVA (ROIS × auditory intensities) for the TW1 showed an effect of auditory intensity. The post hoc analysis showed significant differences between intensity 1 (70.9 dBA) and 5 (94.5 dBA) (*p* = 0.024, Int5 < Int1), nevertheless, the FDR correction did not show any significant difference. For the TW2 the two‐way PERMANOVA, showed similarly an effect of intensity, and the post hoc analysis FDR corrected indicated a difference between the intensities 1 (70.9 dBA), 2 (77.9 dBA), and 4 (89.5 dBA) with the intensity 5 (94.5 dBA) (*p* = 0.007, Int5 < Int1; *p* = 0.006, Int5 < Int2; *p* = 0.014; Int5 < Int4), and also of the intensities 1 (70.9 dBA), and 4 (89.5 dBA) with the intensity 3 (84.5 dBA). (*p* = 0.044, Int3 < Int1; *p* = 0.037, Int3 < Int4). Figure 11 displays the median, mean, and a rain cloud plot, for the ΔHbT significant factors Intensity in the second time window, showing that decrease in the intensity 5 (94.5 dBA).

**FIGURE 12:**
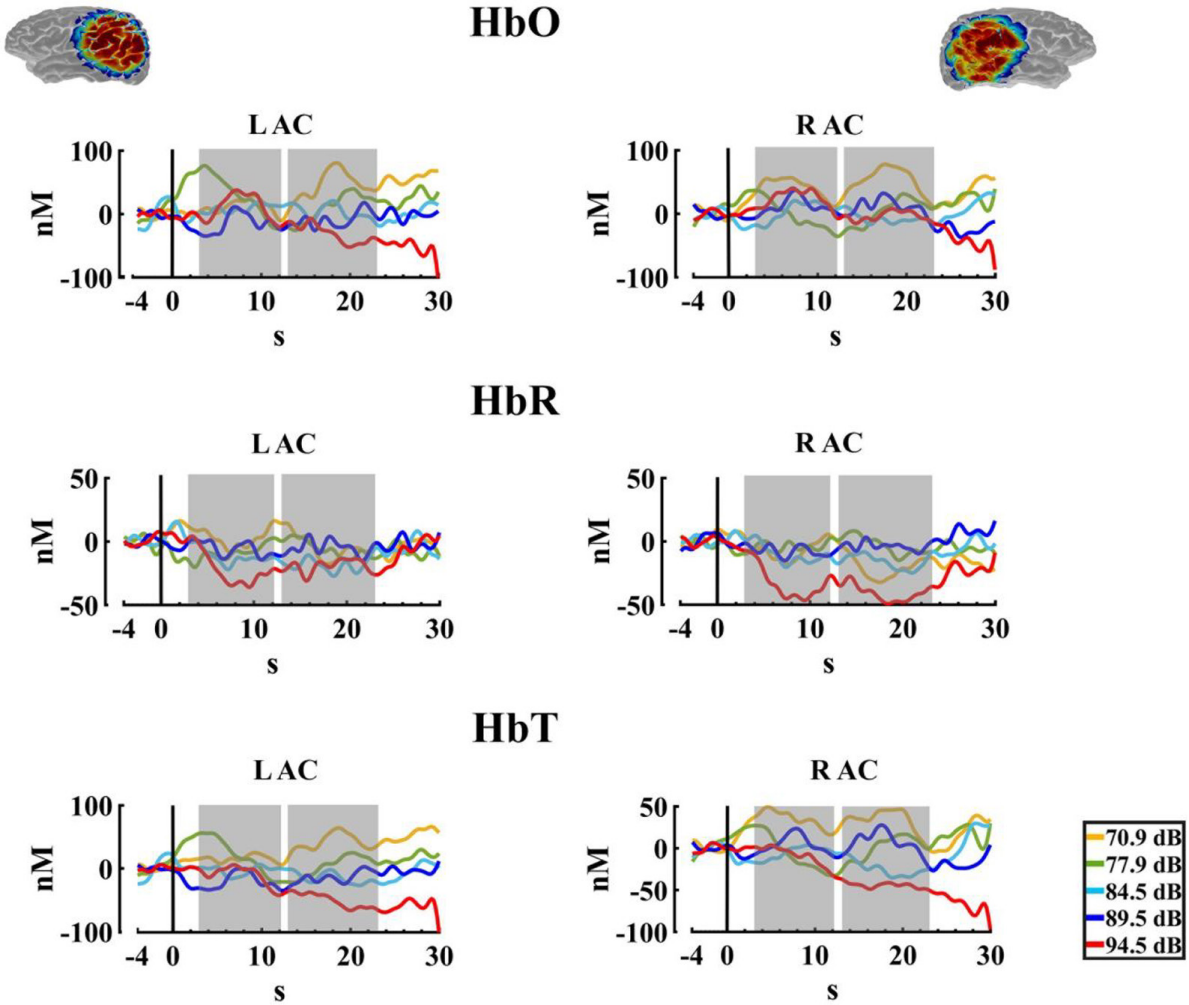
Changes in oxy, deoxyhemoglobin, and total hemoglobin after subtraction of the regressed visual channel activity. Hemodynamic response for ΔHbO, ΔHbR, and ΔHbT, for the five auditory intensities (70.9, 77.9, 84.5, 89.5, and 94.5 dBA), with the values of the regression of the visual channel activity vs auditory cortex activity subtracted from the fNIRS activity in left auditory cortex (LAC) and right auditory cortex (RAC). The sensitivity maps obtained with the fNIRS montage, as indicated by AtlasViewer software, are inserted.

**FIGURE 13:**
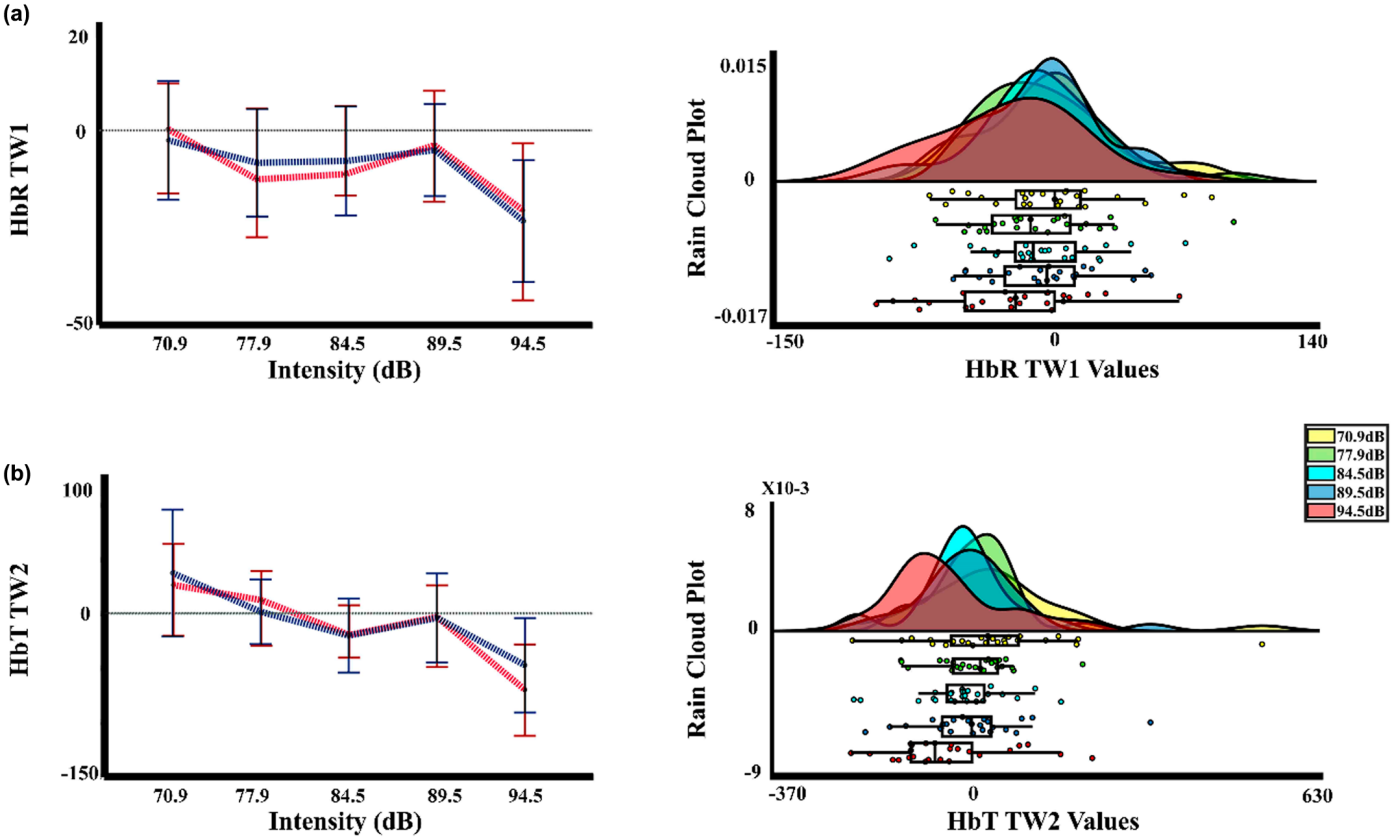
Median, mean values, and a rain cloud plot of the median values for the significant deoxyhemoglobin with regression. (a) ΔHbR in the first time windows with the collapse of the ROIS, for the five auditory stimuli, and (b) ΔHbT in the second time window, with the ROIS collapsed, for the five auditory stimuli. Median: red, mean: blue.

Finally, to eliminate the influence of the common head global activity (both peripheral scalp and brain central) we performed a regression of the visual channel to the signal, subtracting the regressed signal values of the visual channel from the signal values of the right and left auditory cortex. For the data processing, the same parameters were established in Homer2, and the statistical analysis performed for this data was also the same, as described previously. Figure 12 shows the response for the auditory left and right cortices in three types of Hb. The two‐way PERMANOVA (ROIS × auditory intensities), just showed an effect of intensity for the ΔHbT in the TW2. The post hoc analysis showed differences between the intensity 1 (70.9 dBA), and 2 (77.9 dBA) with the intensity 5 (94.5 dBA) (*p* = 0.027, Int5 < Int1; *p* = 0.019, Int5 < Int2), but the FDR correction did not show any significant difference. Likewise, without being significant, the two‐way PERMANOVA analysis found a trend for the intensity in the ΔHbR in the TW1. The post hoc not corrected showed a significant difference for the intensities 1 (70.9 dBA) with the intensity 5 (94.5 dBA), showing a decrease in the signal, being the expected response when assuming a cortical activation (*p* = 0.019, Int5 < Int1), but the FDR correction did not show any significant difference between the intensities. Figure 13 shows the median, mean, and a rain cloud plot for the significant factors in the ΔHbR, and ΔHbT, showing despite regression an increase for total hemoglobin and a decrease in deoxygenated hemoglobin, but in different windows.

**FIGURE 14:**
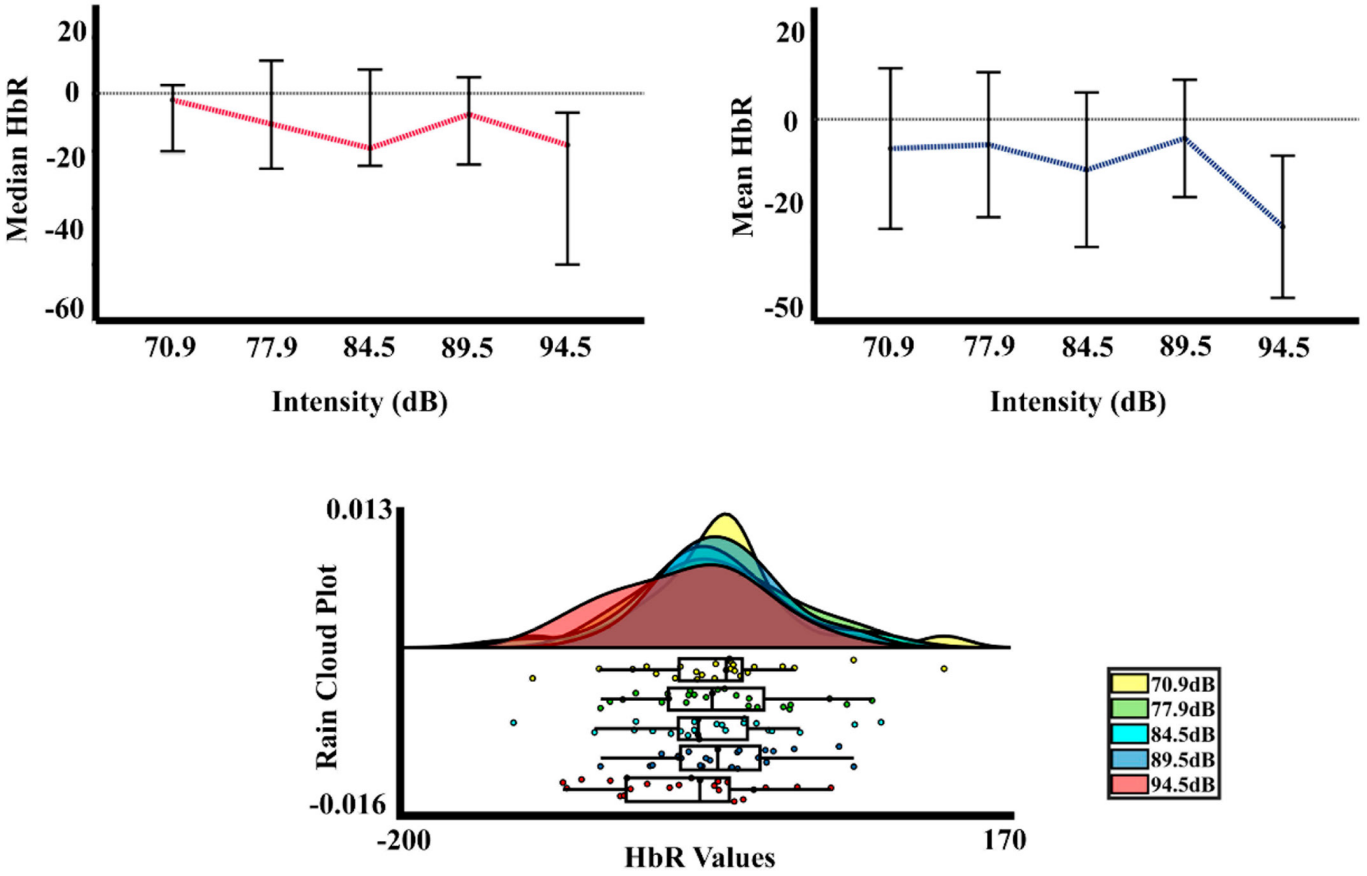
Median, mean values, and a rain cloud plot of the median values for the significant *t*‐test to baseline for deoxyhemoglobin after the regression of the visual channel. ΔHbR for the collapse of both ROIS for the five auditory intensities. Median: red, mean: blue.

To test whether cortical activation to stimulation occurred with respect to baseline after the regression of the visual channel, a *t*‐test FDR corrected comparison was performed, for the HbO, HbR, and HbT signal for each stimulus intensity collapsing hemoglobin concentrations of LAC and RAC. The result showed that intensity 5 (94.5 dBA) was significant with respect to baseline (*p* = 0.025) for HbR, showing a reduction in the concentration for this chromophore (Figure 14).

## 4 DISCUSSION

The results of this study show a systemic response induced by the highest intensity auditory stimulus presented, that is, 94.5 dBA, characterized by a short duration decrease in HR, a decrease in the absolute PPG, and the PTT amplitudes, and an increase in the LFnu power in PPG. At the head level, a decrease in ΔHbO and ΔHbT was found, which replicates the findings of Muñoz‐Caracuel et al. (2021), and which, supported by the decrease in PTT, and increase in LFnu power of PPG, could be explained by a mechanism of arterial and/or arterioles vasoconstriction. However, given that fNIRS short channels were not recorded, the changes at the head level must be interpreted as global: central and/or peripheral. It is important to note that the present study replicates those results showing a decrease not just for the auditory channels, but also in the visual channel, showing a general trend at the head level. However, it is not possible to disentangle if the changes found were due to scalp peripheral and/or cerebral central vasoconstriction. If present fNIRS results are taken together with those of Muñoz‐Caracuel et al. (2021), in which a decrease of HbO and HbT brain concentration after high‐intensity auditory stimulation was obtained, it can be speculatively suggested that vasoconstriction appears at central and peripheral level with high auditory intensity stimulation.

Previous studies of EDA during auditory stimulation paradigms have found an increase in SCR and SCL with auditory stimulation, attributing such changes to a sympathetic control after a stimulus that is perceived as disturbing. Mackersie and Calderon‐Moultrie (2016) found changes in SCL and in general, an increase in psychophysiological reactivity after an auditory demanding task as speech repetition in healthy and normal‐hearing subjects. Other studies (Bakker et al., 2009; Bharath et al., 2020; Chang et al., 2012; Kato et al., 2014; Kishan et al., 2018; Pfeiffer et al., 2019) have analyzed the role of the SNS, through the measurement of skin conductance in children with autism, in anxiety disorders, menopausal women, and auditory hypersensitivity, all of them have related the increase in SCR and/or SCL with the presence of auditory stimulation of different intensities (between 78 and 104 dB). In the present study, although the increase in Mean EDA response was qualitatively evident in the recordings (Figure 2) this measurement was not statistically significant for the intensity factor. However, a previous study in our laboratory (Muñoz‐Caracuel et al., 2021), found a response of increase in this signal with the 94.5 dBA auditory intensity. Likewise, the analysis of the Phasic EDA and SCRs did not show significant results for any of the measures analyzed, neither in the number of SCRs by intensity nor in their mean, integral or maximum value. It should be taken into account that due to the duration of the stimulation and rest periods in the present experiment (50 s), few trials were presented for each auditory intensity which could affect the signal‐to‐noise ratio.

To our knowledge, few studies have been performed with PPG and auditory stimulation (Ooishi & Kashino, 2012). In the literature, the study of this signal has been related to changes in blood volume or BVP (Abay & Kyriacou, 2022; Grote et al., 2003). The PPG signal has been interpreted as oscillations in light scattering and absorption, due to heart‐induced blood flow changes (Nitzan & Zehava, 2022). Nevertheless, the methods for its analysis do not seem to follow a structured guideline. Thus, taking into account our results, and those of Muñoz‐Caracuel et al., 2021, we could claim that the decrease in absolute PPG found at an intensity of 94.5 dB could be related to a decrease in blood volume recorded with PPG, which in turn would support the results found with PTT, being an indication of possible vasoconstriction at high intensity. Regarding its spectral analysis, studies have focused especially on populations with cardiovascular diseases or autonomic regulation (Bernardi et al., 1996; Ishbulatov et al., 2020; Karavaev et al., 2021; Kiselev & Karavaev, 2020). Finding a strong potential in the detection of these diseases with normalized LF values, suggesting that the PPG signal is related to the regulation of peripheral vascular tone, the results of the present study support this hypothesis, since at high intensity (94.5 dB), the LFnu power shows a significant increase, suggesting a vascular tone response, possibly a vasoconstriction process supported by the results of a shorter PTT and decreased mean of the absolute PPG. For the significant results of the LF/HF ratio, there is no physiological explanation, taking into account that in the PPG signal the HF is considered a consequence of the respiration process and not an index of vagal tone as in HRV (Bernardi et al., 1996). Nevertheless, Kiselev and Karavaev (2020), found an increase in LF/HF ratio in patients with hypertension and/or coronary artery disease as compared to healthy subjects, in this sense seems that these patients have an irregular vascular sympathetic activity.

Most studies analyzing the response of the cardiac variability during auditory stimulation have shown inconsistent results, or have found no differences in HRV or cardiac autonomic regulation between sound and no sound conditions (Veternik et al., 2018). Some studies have found differences with different intensities, but the relationship with sympathetic or parasympathetic functions is unclear. A study that presented white noise with intensities of 50, 60, 70, and 80 dB, found that the mean HR, mean arterial pressure, and HF did not change with the presentation of the auditory noise. However, LF and the LF/HF ratio increased with the presentation of the 50‐, 60‐, 70‐, and 80‐dB, apparently, the ANS, especially the SNS, reacted to the noise since the lower intensity. Furthermore, LF was significantly correlated with noise intensity, suggesting greater sympathetic activation by higher noise intensity (Lee et al., 2010). In addition, a study with heavy metal and baroque music that used different intensities (60–70, 70–80, and 80–90 dB), showed that the style of music presented influences the HRV, the baroque music did not alter the cardiac autonomic responses, but heavy metal music in the 60–70 and 80–90 dB reduced the global component of the HRV, possibly explained as an increase in sympathetic modulation leading to a reduction in HRV associated with a high‐intensity sound (do Amaral et al., 2015).

The present study did not find a relationship between sound intensity and HRV in the frequency domain just a trend for the aHF power, however, it should be noted that our experimental design had a short time window of 50‐s post‐stimulus, which limits the results obtained in the time and frequency domain, Therefore, a study with a longer time window could clarify the effects of HRV with sound intensity. It is also important to note that currently there is a debate on the assumption of the low frequency as a reflection of sympathetic activity since in these frequencies a contribution of the parasympathetic component has also been found (Ernst, 2017; Reyes del Paso et al., 2013). In this sense currently, some studies have focused on the analysis of nonlinear measurements of HRV, taking into account the complex dynamics of the ANS and their branches, and the interplay with other physiological subsystems, giving, as a result, a complex and nonstationary cardiovascular dynamic. This analysis includes studies of complexity, entropy, time‐varying analysis, or QT‐variability index, and currently are proving to be more reliable, especially in the detection of cardiovascular disease (Sharif et al., 2016; Valenza et al., 2017). However, the reliability of distinguishing between sympathetic and parasympathetic involvement is still weak. For the time domain a difference in the RMSSD was found, but interestingly, and opposing to the other metrics results, this difference was found for the intensity 3 (84.5 dB), is important to take into account that the RMSSD has been related to parasympathetic tone, in this sense seems that the regulation to this low intensity could be mediated by vagal tone. However, as with frequency domain analysis, its sympathetic or parasympathetic effect is still debated (Kuusela, 2012).

Following the cardiac results, a decrease of HR to sound intensity, in the 94.5 dBA condition was found in the first 3‐s post‐stimulus. These results replicate the findings of Muñoz‐Caracuel et al. (2021), and other studies (Chou et al., 2014; Dykman & Gantt, 1956; Knippenberg et al., 2012; Wilson, 1964; Zeaman et al., 1954) which have found a deceleration in a few seconds time window post‐stimulus with high auditory stimulation, possibly related to the attention or orientation reflex toward a potential threat, some authors describe this effect as an increase in parasympathetic activity. However, According to Uijtdehaage and Thayer (2000), the SNS and PNS are tonically active showing complex interactions, and the cardiac effect shown could be given by a sympathetic and parasympathetic interaction. Similarly, the debate about whether deceleration is a defense reflex or not is still unclear. Nevertheless, considering our experimental design, the present study is not intended to be included in this debate, but to show broadly a systemic reaction to auditory stimulation at different levels of intensity, that in the HR shown a decrease at the highest intensity level.

Interestingly, contrary to what was previously found in some studies with fNIRS (Bauernfeind et al., 2018; Chen et al., 2015; Ehlis et al., 2009; Weder et al., 2018; Weder et al., 2020) and fMRI (Brechmann et al., 2002; Hall et al., 2001; Hart et al., 2003; Langers et al., 2007; Röhl & Uppenkamp, 2012; Sigalovsky & Melcher, 2006), our results suggest a decrease for all the chromophores at the brain level at an intensity of 94.5 dBA, replicating the results of Muñoz‐Caracuel et al. (2021), who found the same pattern of response at the same level of intensity, in this case, is interesting that the results are accentuated (with lower *p* values) in the second time window (13–23 s) showing a decrease at the head level that extends over time and is more pronounced. It could be expected that since it is an auditory stimulation paradigm, the auditory cortex would show a higher level of oxygenation reflecting activation in response to the stimulus, but as proposed by Münzel et al. (2014), the sympathetic response to sound or noise does not always require cortical activation but rather a subcortical activation at the level of the hypothalamus, endocrine and limbic system, which prepares for flight upon a perception of threat. This statement could explain why at the cortical level a pattern of activation response to sound at a higher intensity is not detected.

However, when performing the visual channel regression to discount the global head fNIRS signal (cerebral and scalp peripheral), a likely pattern of activation was found for the HbR chromophore, limited to significant effects of the highest intensity (i.e., 94.5 dBA). The explanation for finding this effect only in HbR chromophore could be due to a higher physiological interference for HbO concentration changes (Pinti et al., 2020). On the other hand, changes in HbR, despite having lower intensity than HbO, are less contaminated by physiological signals and have shown a higher correlation with fMRI results (Kirilina et al., 2012). One study carried out by Steinmetzger et al. (2020), with five different stimulus conditions consisting of modulated like‐speech tones, found no significant activation of the auditory cortex for the HbO, but the HbR data showed the expected activation of the auditory cortex, as well in the present study.

A possible explanation for the signal decrease found at the head (fNIRS) and peripheral levels of blood flow signals (absolute PPG and PTT), could be not just a peripheral, but a systemic vasoconstriction response that could clarify the decrease in oxygenation head level in the snapshot in which the signals are recorded. That decrease in the PTT, and increase in the LFnu power of PPG was supported by data from the Muñoz‐Caracuel experiment. Therefore, sympathetic control could be inferred due to vasoconstriction as a response toward the higher intensity, as a result of a defense reflex to the perceived threat. During vasoconstriction, arterial stiffness increases causing an increase in pulse wave velocity, consequently, the propagation time of a pulse wave from the heart to the peripheral arteries decreases. Some studies have found evidence of vasoconstriction in auditory paradigms using PTT and laser Doppler flowmetry (Feger & Braune, 2005; Franco et al., 2002; Galland et al., 2007; Sato & Ooishi, 2012). Is important to note that the fact that after subtracting the activity of the visual channels from the auditory channels the effect of intensity obtained for the HbT was a concentration decrease, suggesting that the vasoconstriction sympathetic activity induced by high‐intensity auditory stimulation was global and not specific to auditory areas.

On a general level, the results of the present study suggest a systemic and complex activation, given by a response to high‐intensity auditory stimulation being perceived as a threat, and that at the peripheral level is reflected as a response of cardiac deceleration (parasympathetic activity) and vasoconstriction (sympathetic activity) and at the head level as a reduced in all the chromophores concentration probably due to vasoconstriction. These results show a broad and complex pattern of response that in the future can potentially contribute to the study of the defense or orienting reflexes responses in disorders such as autism, anxiety, schizophrenia, or attention‐deficit disorder, in which it is known that the levels of autonomic activation could be affected.

### 4.1 Limitations

Apart from the already described lack of short channel recording, some limitations of the presented study should be taken into account, firstly the results of the HRV could be affected by the short time window in our experimental design (i.e., 50 s), which is too short to estimate frequency components, especially LF. In the same vein, it is known that the HF values are largely affected by respiration rate. On this basis, the use of a longer time window will be taken into account in the future, as well as to include respiration measurements in the procedure. Similarly, the use of more sophisticated techniques not only for signal complexity or time‐varying analysis but also for signal synchronization.

The present report does not take into account the participants' self‐reports of sound loudness, which according to recent research (Weder et al., 2020) explains better the results in the fNIRS signal than the acoustic pressure intensity values. A future study will take this approach into account to improve the results of fNIRS activation, as well as the inclusion of short channels in the montage.

It is important to emphasize that the design of this study was conducted to find a pattern of response at a systemic level both peripheral and central, thus the stimulation protocols have been adapted as best as possible to include all possible measures, however, we are aware that some of the results obtained could vary or even contradict the results obtained with standardized paradigms. For this reason, in the future, we intend a better inclusion of the standard protocols of the different measures to find greater generalizability in the results presented above.

## 5 CONCLUSION

The results found in the present study could indicate the presence of an autonomic systemic response to high sound intensity, that is, 94.5 dBA, possibly generated by a threat response that could produce a decrease in HR, peripheral absolute PPG, PTT, an increase in LFnu power of PPG, and head vasoconstriction (cerebral and/or the scalp) within a few seconds of the presentation of the aversive stimulus. This result provides a potential for its application in a variety of disorders that involve an altered defensive response.

## AUTHOR CONTRIBUTIONS

**Vanesa Muñoz** analyzed the data, made the figures, and drafted the manuscript, **José A. Diaz‐Sanchez and Manuel Muñoz‐Caracuel** carried out the experiments. **Carlos M. Gómez** designed the study and was in charge of review and editing. All authors approved the final version of the manuscript.

## FUNDING INFORMATION

This study was supported by the Spanish Agencia Estatal de Investigación (AEI) (PID2019‐105618RB‐I00) (FEDER funds), by the Consejería de Innovación, Ciencia y Empresa of the Junta de Andalucía (P20_00537) and Vanesa Muñoz was funded by a scholarship by Universidad de Sevilla (VIPPIT‐2020‐IV.3).

## CONFLICT OF INTEREST

The authors have no relevant financial or nonfinancial interests to disclose.

## Supporting information




Figure S1
Figure S2
Figure S3
