# Supplementary figures and images for: Head hemodynamics and systemic responses during auditory stimulation
Source: Physiol Rep. 2022 Jul 3;10(13):e15372. doi: 10.14814/phy2.15372 (PMC9251853; doi:10.14814/phy2.15372)

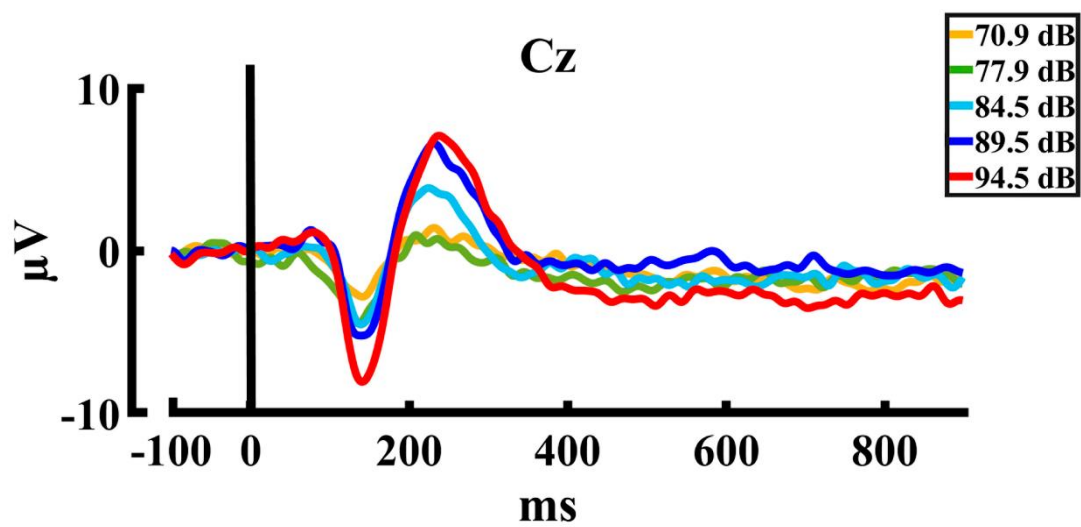

Supplementary Figure 1.

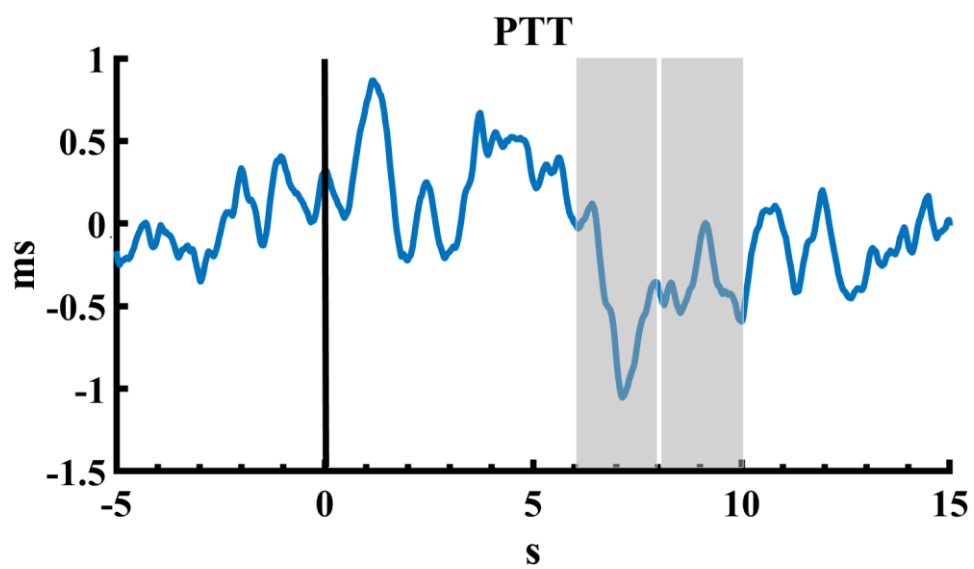

Supplementary Figure 2.

A)

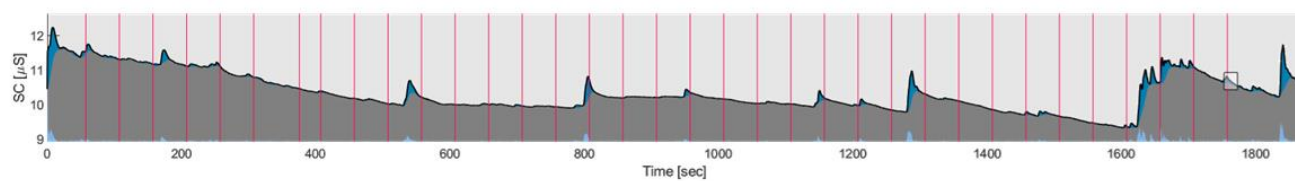

B)

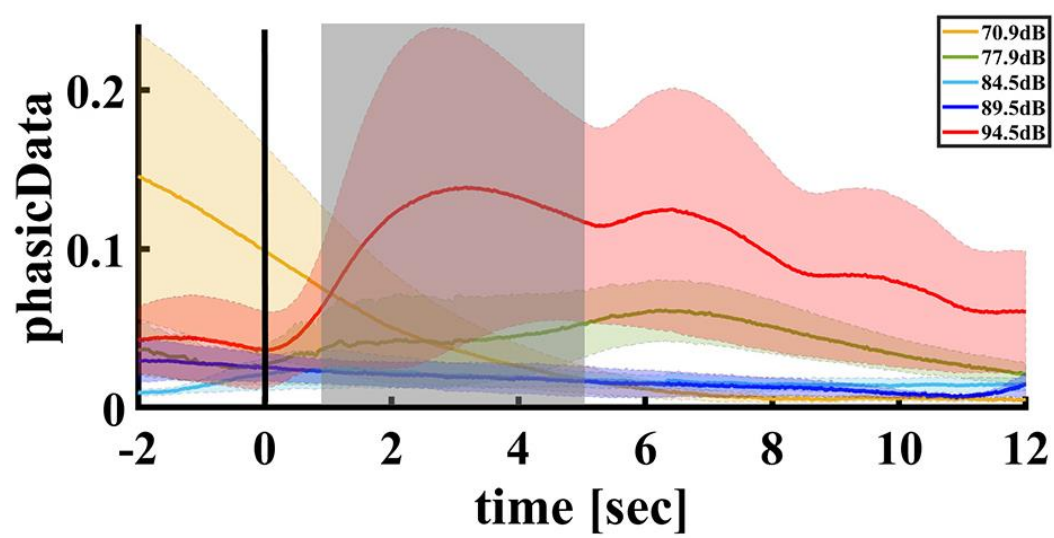

Supplementary Figure 3.

Supplement: Supplementary file 1 — Figure S1 Figure S2 Figure S3 [file PHY2-10-e15372-s001.pdf]
